# Engineering exosomes derived from subcutaneous fat MSCs specially promote cartilage repair as miR-199a-3p delivery vehicles in Osteoarthritis

**DOI:** 10.1186/s12951-023-02086-9

**Published:** 2023-09-22

**Authors:** Shu Zhao, Guanghui Xiu, Jian Wang, Yi Wen, Jinyuan Lu, Baitong Wu, Guangming Wang, Danjing Yang, Bin Ling, Dajiang Du, Jun Xu

**Affiliations:** 1 East Hospital, School of Medicine, Tongji University, Shanghai, People’s Republic of China 200120; 2 Department of Plastic Surgery, Shanghai Fourth People’s Hospital, School of Medicine,Tongji University, Shanghai, 200434 People’s Republic of China; 3 Department of Hematology, Tongji Hospital, School of Medicine, Tongji University, Shanghai, China; 4 Institute of Microsurgery on Extremities, and Department of Orthopedic Surgery, Shanghai Sixth People’s Hospital Affiliated to Shanghai Jiao Tong University School of Medicine, Shanghai, 200233 People’s Republic of China; 5 Department of Intensive Care Unit, Affiliated Hospital of Yunnan University (The Second People’s Hospital of Yunnan Province), Yunnan University, Kunming, 650021 People’s Republic of China

**Keywords:** Engineering exosomes, Osteoarthritis, Autophagy, miR-199a-3p, Cartilage-targeted

## Abstract

Osteoarthritis (OA) is a degenerative joint disease involving cartilage. Exosomes derived from Mesenchymal stem cells (MSCs) therapy improves articular cartilage repair, but subcutaneous fat (SC) stromal cells derived exosomes (MSCs^SC^-Exos), especially engineering MSCs^SC^-Exos for drug delivery have been rarely reported in OA therapy. This objective of this study was to clarify the underlying mechanism of MSCs^SC^-Exos on cartilage repair and therapy of engineering MSCs^SC^-Exos for drug delivery in OA. MSCs^SC^-Exos could ameliorate the pathological severity degree of cartilage via miR-199a-3p, a novel molecular highly enriched in MSCs^SC^-Exos, which could mediate the mTOR-autophagy pathway in OA rat model. Intra-articular injection of antagomiR-199a-3p dramatically attenuated the protective effect of MSCs^SC^-Exos-mediated on articular cartilage in vivo. Furthermore, to achieve the superior therapeutic effects of MSCs^SC^-Exos on injured cartilage, engineering exosomes derived from MSCs^SC^ as the chondrocyte-targeting miR-199a-3p delivery vehicles were investigated in vitro and in vivo. The chondrocyte-binding peptide (CAP) binding MSCs^SC^-Exos could particularly deliver miR-199a-3p into the chondrocytes in vitro and into deep articular tissues in vivo, then exert the excellent protective effect on injured cartilage in DMM-induced OA mice. As it is feasible to obtain human subcutaneous fat from healthy donors by liposuction operation in clinic, meanwhile engineering MSCs^SC^-Exos to realize targeted delivery of miR-199a-3p into chondrocytes exerted excellent therapeutic effects in OA animal model in vivo. Through combining MSCs^SC^-Exos therapy and miRNA therapy via an engineering approach, we develop an efficient MSCs^SC^-Exos-based strategy for OA therapy and promote the application of targeted-MSCs^SC^-Exos for drug delivery in the future.

**Graphical Abstract:**

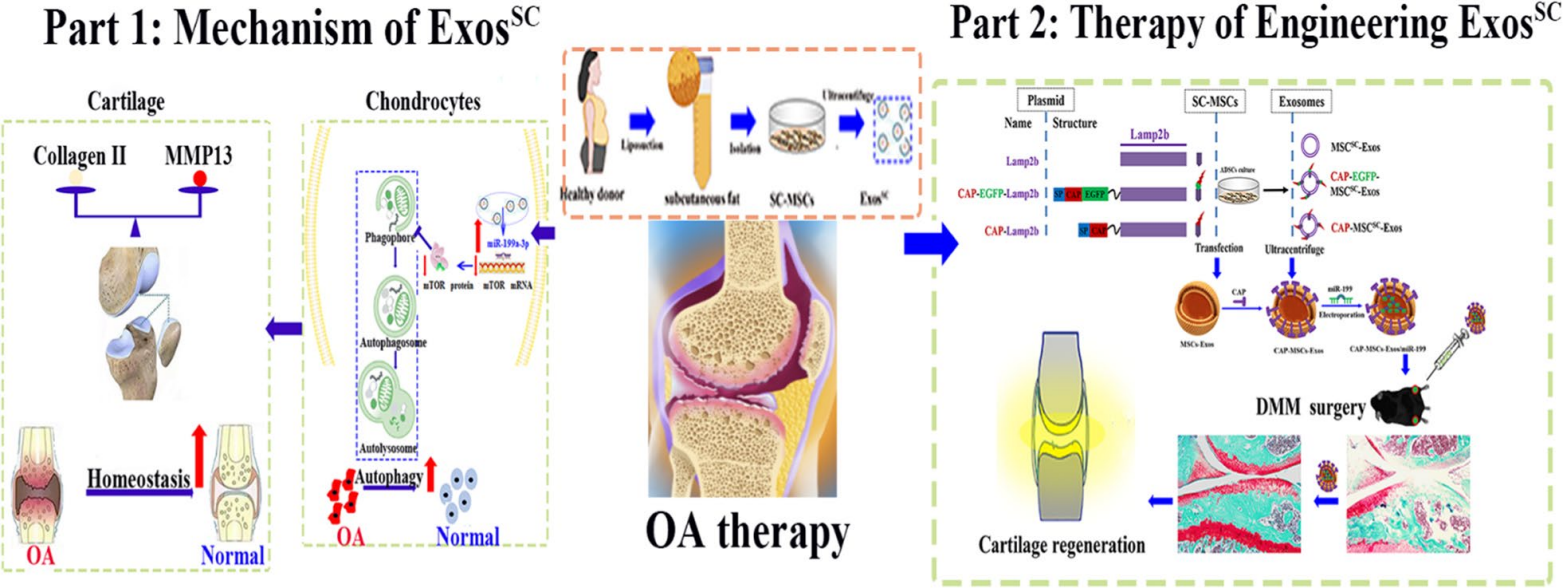

**Supplementary Information:**

The online version contains supplementary material available at 10.1186/s12951-023-02086-9.

## Introduction

Osteoarthritis (OA) is a common and chronic degenerative joint disease, which causes severe pain and substantial economic burdens. OA is now recognized as a mild inflammatory disorder that affects the entire joint, particularly successive breakdown of cartilage, abnormal chondrocyte hypertrophy, inflammation of synovium, and disturbances of the tidemark preceded via angiogenesis occurred at the osteochondral junction [1–3]. The impaired chondrocytes homeostasis is vital induction of OA [4, 5]. It is urgent to explore effective and safe treatments for OA, mainly repairing the damaged cartilage. Recently, mesenchymal stem cells (MSCs) have entered clinical trials, and allogenic MSCs are demonstrated to be safe and less invasive therapy for OA patients [6–9]. Various source of MSCs like bone marrow-derived MSCs, adipose tissue derived MSCs and umbilical cord-derived MSCs are capable of alleviating cartilage destruction, due to their immunomodulatory and regenerative capacities [10, 11]. Adipose-derived stromal cells (ADSCs), with more approachable and plentiful acquisition, are not only possess the fundamental features of MSCs, but also presented powerful ability of cartilaginous proliferation compared to other types of MSCs [12]. According to mounting evidence, MSCs-based treatment efficiency on cartilage regeneration depends on paracrine signaling, including secreted factors and extracellular vesicles (EVs). Exosomes (Exos) are one subtype of extracellular vesicles with a diameter ranging from 40 to 150 nm, secreted by almost all types of cells [13]. Exosomes serve as a novel intercellular shuttle, by transferring various biomolecules, including lipids, cytosolic proteins and nucleic acids (predominantly microRNAs) between cells [14]. MSCs-Exos are much lower immunogenicity, convenient procession, higher stability and simple storage, comparing to MSCs [12]. Preliminary results showed MSCs-Exos could promote the repair of degenerated cartilage along with synthesis of type II collagen and ECM, including: embryo-derived MSCs [15], umbilical cord-derived MSCs [16], bone marrow-derived MSCs [17] and adipose tissue derived MSCs [18–20]. Taken together, MSCs-Exos are used as a cell-free treatment for treating OA diseases.

MSCs-Exos, which are derived from adipose tissue especially subcutaneous fat (SC), are easily collective by liposuction from healthy donors and belong to surgical waste. Our previous research had shown the effective therapy of SC-derived MSCs and MSCs^SC^-Exos in motor neuron disorders [2, 21], tissue damage illness [22, 23], stroke syndromes [24] and hepatic fibrosis [25]; indicating that MSCs^SC^-Exos would be a potential treatment for cartilage degeneration. To the best of our knowledge, MSCs^SC^-Exos function in cartilage protection have not been thoroughly investigated. Only one research presented that MSCs^SC^-Exos mitigated cartilage degradation by transferring miRNA-376c-3p and targeting the Wnt-bet-catenin signaling axis in MIA-induced OA model [20]. It is well known that the MIA model develops rapid lesions, which do not represent the chronic progression of human OA disease [26]. Therefore, the underlying mechanism of SC-derived MSCs-Exos protecting the cartilage remains obscure, and need to be in-depth clarified.

Exosomes have emerged as new drug delivery systems accompany with quantities of academic researches and industrial development in the recent years [27]. Due to their biological activity, low immunogenicity, intrinsic biocompatibility and targeting capability, loading exosomes with exogenous therapeutic drug/nanoparticles is practicable, especially the surface protein or lipid membrane of exosomes through biological engineering and chemical engineering to develop the cell-specific drug delivery systems [28–30]. The drug delivery systems based on exosomes from subcutaneous fat (SC) derived MSCs with easier accessibility and high performance, seem a valuable strategy. Up to now, the majority of the researches were focused on the native exosomes derived from MSCs^SC^ as innovative medicine, while the current research of the engineer exosomes derived from MSCs^SC^ for drug delivery is still limited [27]. In the aspect of cartilage targeting exosomes, only one study has reported that engineering exosome with CAP peptide derived from murine dendritic cells specifically targeted chondrocytes, and showed more pronounced therapeutic effects in a rat OA model than non-tagged exosomes [31]. The current study about MSCs^SC^-Exos as the engineering exosomes of cartilage targeting is still blank. Furthermore, to achieve the superior therapeutic effects on injured cartilage, engineering exosomes derived from MSCs^SC^ that are fusing an chondrocyte-binding peptide CAP with the exosomal membrane protein Lamp 2b were created, and were investigated in the DMM induced OA mice.

In this research, we aimed to explore the therapeutic potential of SC-derived MSCs-Exos on destabilization of the medial meniscus (DMM) and anterior cruciate ligament transection (ACLT) induced OA disease, which are more representative of human OA [32, 33] and examine if the transmission of miRNA from MSCs^SC^-Exos to chondrocytes played an important role in cartilage injury. Our results indicated that MSCs^SC^-Exos could ameliorate the pathological severity degree of cartilage via miR-199a-3p-mediated mTOR-autophagy pathway in the OA rat model. Furthermore, to achieve the superior therapeutic effects on injured cartilage, engineering exosomes derived from MSCs^SC^ that are fusing an chondrocyte-binding peptide CAP with the exosomal membrane protein Lamp 2b were designed to realize chondrocyte-targeted delivery of miR-199a-3p. Intra-articular injection of chondrocytes-targeted MSCs^SC^-Exos loaded with miR-199a-3p has presented significant cartilage regeneration in DMM induced OA mice. Above all, a valuable and efficient cell-free therapy based on engineering exosome derived from MSCs^SC^ is highly recommended for cartilage regeneration and OA therapy.

## Methods

### Preparation of SC-derived MSCs, primary articular chondrocytes and  synovial cells

The subcutaneous adipose tissue was obtained by liposuction from young healthy donors. The methods and guidelines were approved by the People’s Liberation Army No. 85 Hospital, Shanghai, P.R. China (review serial number NO.2013/18) [34]. All donors provided written informed permission. All operation was conducted in accordance with the regular procedures and Declaration of Helsinki.

SC-derived MSCs was isolated in accordance with the earlier reported reference [25]. After washed with phosphate-buffered solution (PBS) (Gibco, Rockford, IL, USA), 500 mg of adipose tissue cut into 1-mm^3^ pieces was digested for 45 min with 0.1% collagenase I (Gibco, USA). Then, we added equivalent volume of complete culture medium (DMEM-F12 culture medium (Gibco, USA), 10% FBS, 1% Penicillin–Streptomycin) and centrifuged at 1000 rpm for 10 min. cells were cultured at a 1 × 10^6^/ml density in complete culture medium with 10 ng/ml bFGF (Stem Cell, USA) after being resuspended in PBS and filtered using a 40 μm cell strainer. When adhering cells reach 80–90% confluence, the cells should be passaged in a 1:3–1:4 ratio. The primary SC-derived MSCs before passage 7 were used.

The surface markers of MSCs^SC^ were identified according to the experiment design of the Human MSC Analysis Kit (BD Biosciences) and by using flow cytometry (FACSCalibur, BD, NJ, USA). Data was assessed with Flow Jo V10 software.

The primary articular chondrocytes was isolated according to the earlier research [35]. The cartilage samples from newborn Sprague-Dawly (SD) rats was rinsed in sterile PBS contained penicillin–streptomycin and then sliced. The matrix was digested overnight in high-glucose DMEM (Gibco, USA) with 0.2% type II collagenase (Gibco; 17101-015) and 1% P/S to extract cartilage. The cell solution was filtered through a 70 μm cell strainer; and the collected primary chondrocytes were centrifuged at 400 g for 5 min before resuspending in high-glucose DMEM supplemented with 10% FBS and 1% P/S. The medium was exchanged every next day. The primary chondrocytes before passage 4 were used. C28/I2 cells, which are the normal human chondrocytes, were cultured in DMEM (Gibco, USA) with 1% P/S and 10% FBS (Gibco, USA) in a 5% CO_2_ environment at 37 °C.

The primary synovial cells was isolated as described previously [36]. The synovial tissue samples from 8-week old SD rats was rinsed in sterile PBS contained P/S and then sliced. The matrix was digested in RPMI medium (Gibco, USA) with collagenase (2 mg/ml) (Sigma, C5138, St. Louis, MO) and 1% P/S at 37 °C for 1 h. The cell solution was filtered through a 70 μm cell strainer; and the collected primary synovial cells were centrifuged at 400*g* for 5 min before resuspending in RPMI medium supplemented with 10% FBS and 1% P/S. The medium was exchanged every next day.

### Isolation and identification of Exos derived from MSCs^SC^

To obtain the MSCs^SC^ derived exosomes, when MSCs^SC^ on the 2nd passage neared 80–90% confluence, the DMEM-F12 culture medium added with 10% exosome-depleted serum and 10 ng/ml bFGF were replaced for 24 h, after that the cell supernatant was all collected. The cell supernatant was pre-centrifuged in 300*g* for 10 min, 2000*g* for 20 min, 10,000*g* for 30 min to eliminate dead cells, apoptotic bodies and cell debris. Then, after 0.22 μm filtration, the whole supernatant was moved to ultracentrifuge tube (Beckman, #355618, USA) and ultracentrifuged in 100,000*g* for 70 min twice to separate exosomes. The pellets were then resuspended in 100 μl PBS per tube. All operations were performed at 4 °C and the generated exosomes were stored at − 80 °C or used instantly.

The Nanoparticle Tracking Analysis (NTA) was used to measure exosomes concentration, the distribution of particle size and purity (NanoSight300, Malvern Instruments Ltd, UK). 1 ml PBS was used to dilute the samples with the appropriate concentration before being put into the sample cubicle. The particles were then monitored and illuminated via Brownian motion of laser light. Each sample was performed three times, and the procedure was documented. The Stokes–Einstein equation was used to calculate the concentration of particles, the distribution of size and scatter intensity.

The transmission electron microscopic (TEM) technique was employed to identify the ultrastructure of the exosomes. Hitachi HT7800 electron microscope (HT-7800, Hitachi, Japan) was used to capture images of MSCs^SC^-Exos.

### Plasmid construction and transfection

The pEGFP-C1-RVG-Lamp2b expressing vector was kindly provided by Prof. Matthew J. A. Wood’s lab from University of Oxford [37]. The DNA sequence encoding a glycosylation motif (GNSTM) [38], CAP Peptide (DWRVIIPPRPSA) [39], and a glycine-serine spacer were synthesized and subcloned to replace the RVG fragment in the plasmid vector, then was refined as plasmid CAP-Lamp2b. Meanwhile, the other two plasmid encoding Lamp2b and CAP-EGFP-Lamp2b, were constructed respectively, according to the previous report [31]. The above three plasmids encoding the lamp2b constructs were transfected into the SC-derived MSCs using Lipofectamine 3000 transfection reagent (Invitrogen, L3000008, USA), when adhering cells reach 50–60% confluence. The corresponding exosomes were isolated according the previous method in 2.2.

### In vivo studies

#### Ethics statement

The animal experiments were approved by Ethical Committee of Laboratory Animals Research Center, Tongji University. The approval number was TJAA07622701. All experimental procedures on procedures on animals were carried out in accordance with the Guidelines for the Care and Use of Laboratory Animals of the National Institutes of Health.

#### Rats OA model and grouping

Six-week-old male SD rats about 180–200 g were purchased from SLAC Laboratory Animal Co. Ltd. (Shanghai, China). All rats were maintained on a 12-h light cycle in the animal facility of the Animal Unit of Tongji University. When the rats were eight weeks old, they were subjected to destabilization of the medial meniscus (DMM) and anterior cruciate ligament transection (ACLT) surgery to induce OA, as previously described [40]. Specifically, the DMM + ACLT surgery was performed by surgical sectioning of the medial meniscotibial ligament and the sham operation was performed by incision of the cutaneous and muscular planes at baseline.

In the first animal section (Fig. 2B), all rats underwent DMM + ACLT surgery or sham surgery were randomly divided into three groups: (1) sham group; (2) PBS group; (3) PBS-Exos^SC^ group. Three weeks after operation, rats were given multiple intra-articular injections of 50 μl PBS or 50 μl Exos^SC^ (2 × 10^10^ particles/ml) during the following 6 weeks (once a week). After 8 weeks of operation, anaesthesia of the rats was maintained with isoflurane and the samples were subjected to pathological analysis.

In the second animal section (Fig. 6A), all rats underwent DMM + ACLT surgery or sham surgery were randomly divided into four groups: (1) sham; (2) PBS + antagomir-NC group; (3) PBS-Exos^SC^ + antagomir-NC group; (33) PBS-Exos^SC^ + antagomir-199-3p group. The rats were pre-injected with 50 μl antagomir-NC or antagomir-199-3p for 3 weeks (twice a week), 2 week after surgery; and then the rats were given multiple intra-articular injections of 50 μl PBS or 50 μl Exos^SC^ (2 × 10^10^ particles/ml) during the following 6 weeks (once a week). After 11 weeks of operation, anaesthesia of the rats was maintained with isoflurane and the samples were subjected to pathological analysis.

#### Mice OA model and grouping

Seven-week-old male C57BL/6 J mice about 18–22 g were purchased from SLAC Laboratory Animal Co. Ltd. (Shanghai, China). All mice were maintained on a 12-h light cycle in the animal facility of the Animal Unit of Tongji University. When the rats were nine weeks old, they were subjected to DMM to induce OA, as previously described [40]. The sham group were operated was by incision of the cutaneous and muscular planes at baseline. All mice underwent DMM surgery or sham surgery were randomly divided into five groups: (1) sham group; (2) OA + PBS group; (3) OA + Exos^SC^ group, (4) OA + Exos^SC^/mir-199a-3p group; (5) OA + CAP-Exos^SC^/mir-199a-3p group. Six weeks after operation, mice were given multiple intra-articular injections of 10 μl PBS or 10 μl Exos^SC^ (1 × 10^10^ particles/ml) during the following 4 weeks (once a week). After 10 weeks of operation, anaesthesia of the mice was maintained with isoflurane and the samples were subjected to pathological analysis.

#### The tissues preparation, safranin O/fast green staining trials and immunohistochemical (IHC) trials

The testing animals were killed and the entire knee joints were preserved in 4% paraformaldehyde for 24 h before being decalcified in 0.5 M EDTA at pH 7.4 for 40 days (rats)/7 days (mice) and then being embedded in paraffin. Sections of 5 μm thickness were cut for Safranin O/Fast Green staining and immunohistochemical analysis.

The Osteoarthritis Research Society International (OARSI)-modified Mankin criteria were used to grade the cartilage deterioration in Safranin-O/Fast Green-stained specimens [41]. Two independent, blindfolded graders evaluated each section, and the statistical analysis was based on the average score.

For IHC analysis, the trial sections were incubated overnight at 4 °C with primary antibodies specific for COL2A1 (Proteintech, 28459-1-AP, China), MMP13 (Proteintech, 18165-1-AP). The DAB chromogen kit (Servicebio, G1212, Wuhan, China) was used to visualize the staining of sections. The number of positive antigen-stained chondrocytes and the corresponding total number of chondrocytes was calculated, through using Image-Pro Plus 6.0 software (Media Cybernetics, USA). The percentage of positive antigen-stained chondrocytes in different sections and the relative fold changes to the sham group were compared.

#### The tissue immunofluorescence trial

Following decalcification, the joint tissues were rinsed three times with 1 × PBS for 5 min before submerging in 30% sucrose for 12-16 h at 4 °C until the tissues sank. Transmit the tissues to an OCT-containing cryomold (Sakura, 4583, USA), and preserve at − 80 °C. For tissue immunofluorescence, 5 μm thick sections were cut using a freezing microtome (Leica, CM1950, Germany).

The specimen was rinsed three times in chilled PBS for 5 min each before being blocked with blocking buffer [1X PBS (BI, 02-024-1ACS)/5% normal serum (Jackson lab, 005-000-121, USA)/0.3% Triton^™^X-100 (Sangon Biotech, A110694, Shanghai, China)] for 60 min at room temperature. After incubating the specimen with the appropriate antibodies overnight at 4 °C, they were washed three times in PBS for 5 min each. Then specimen was treated for 1 h with matching fluorochrome-conjugated secondary antibody diluted in antibody dilution buffer protected from light, and rinsed three times in PBS for 5 min each. After staining with DAPI (Sigma, 32670), each specimen was rinsed three times for 5 min in PBS. Photographs of the representative images were taken with a confocal microscope laser scanning (Leica, TSC SP8). The mouse anti-COL2A1 (Invitrogen, MA5-12789), mouse anti-MMP13 (Invitrogen, MA5-14238), LC3B-antibody (CST, 43566, Danavers, MA), mTOR-antibody (CST, 2983) was employed as e primary antibodies. For secondary reactions, Alexa Fluor 488 goat anti-mouse IgG secondary antibody (Invitrogen, A32723) and Alexa Fluor 594 goat anti-rabbit IgG secondary antibody (Invitrogen, A32754) were used.

#### Transmission electron microscopy (TEM) assay for animal cartilages

After decalcification, the tissues were subjected to transmission electron microscopy (TEM) assays. The cartilage samples were fixed in 2.5% glutaraldehyde for at least 4 h at room temperature, rinsed with 0.1 M phosphate buffer four times for 15 min each. Then the samples were post-fixed with 1% osmic acid for 1–2 h at room temperature, dehydrated using an ascending acetone, embedded in Epon 812. The samples were sliced into 70-nm-thick sections and were photographed with the JEOL JEM-1230 electron microscope.

#### Sequencing of tissues RNA

Cartilages specimens of Control group (OA + PBS) and MSCs^SC^-Exos treatment group (OA + Exos^SC^) rats were gathered (n = 3/group). The total RNA was then extracted as described above. LC Bio Technology CO., Ltd (Hangzhou, China) sequenced the RNA libraries using the illumina Novaseq^™^ 6000 platform. The bioinformatic analysis, including differentially expressed genes (DEGs), KEGG enrichment, GSEA enrichment was carried out using the OmicStudio tools at https://www.omicstudio.cn/tool. The heatmap was drawn based on the R (https://www.r-project.org/) on the OmicStudio platform (https://www.omicstudio.cn/tool).

### In vitro studies

#### The co-culture assay

The rat chondrocytes were pretreated with *IL-1β* for 24 h and resuspended at a density of 1 × 10^5^ cells/well (2 ml) and then seeded in the lower chambers (6-well migration chambers, 0.4 μm pore membrane, 83.3930.041, SARSTEDT, Germany). The upper chambers were seeded with rat synovial cells of 1 × 10^5^ cells/well in 6-well plates filled with DMEM/F12 medium supplemented with 10% FBS, and incubated at 37 °C with 5% CO_2_ and a humidified incubator. Subsequently, the exosomes (1 × 10^9^ particles/ml) were added in the upper cell culture medium. After 48–72 h incubation, the lower and upper cells were harvested for mRNA and protein analysis, IF and autophagic flux detection.

#### Cell viability assay

The rat chondrocytes were pre-seeded at a density of 4000 cells/well in 96-well plates and cultured overnight. After the administration of MHY1485 (MedChemExpress, Shanghai, China), cell proliferation ability of the rat chondrocytes was evaluated by cell counting kit-8 (CCK-8, Beyotime, China). For different treatments of different doses or times, the OD450 values of cells were measured using microplate reader (SpectraMax M5, Molecular Devices, SanJose, USA).

#### The cell immunofluorescence trial

The chondrocytes staining was performed following a standard protocol. Briefly, chondrocytes were rinsed three times in cool PBS for 5 min each, fixed in paraformaldehyde (4%) or methanol for 20 min, and then blocked with Blocking Buffer for 60 min at room temperature. The chondrocytes were then incubated with the indicated antibodies overnight at 4 °C, and rinsed three times in PBS for 5 min each. Then chondrocytes were incubated with corresponding fluorochrome-conjugated secondary antibody diluted in antibody dilution buffer for 1 h protected from light, and rinsed three times in PBS for 5 min each. The samples were stained with DAPI (Sigma, 32670), and then rinsed three times in PBS for 5 min each. Representative images were photographed using a laser scanning confocal microscope (Leica, TSC SP8). The primary antibodies used were mouse anti-COL2A1 (Invitrogen, MA5-12789), mouse anti-MMP13 (Invitrogen, MA5-14238). And the Alexa Fluor 488 goat anti-mouse IgG secondary antibody (Invitrogen, A32723) were used as for secondary reactions. Four random fields of each group were captured and used for statistical analysis.

#### Western blot analysis

The cartilage and chondrocytes were treated with the RIPA lysis buffer (Epizyme, PC101, China) added with a protease inhibitor cocktail (Epizyme, GRF101, China) and a phosphatase inhibitor cocktail (Epizyme, GRF102). The protein contents were calculated using a BCA protein assay kit (Takara, T9300A), and the samples was instantly boiled for 10 min with the addition of loading buffer. An equal quantity of protein extracts (20 μg) was placed onto a sodium dodecyl sulfate–polyacrylamide gel electrophoresis (SDS-PAGE) gel for electrophoresis and transmitted to a PVDF membrane. Following that, the PVDF membrane was sequentially incubated with primary and secondary antibodies. Finally, the enhanced chemiluminescence (BI, 20-500-120) was used to react with secondary antibodies and the images were acquired. The mTOR-antibody (CST, 2983), P-p70S6 (CST, 9234), Calnexin-antibody (Abcam, ab133615, USA), CD9-antibody (Abcam, ab263019), CD81-antibody (Abcam, ab109201), TSG101-antibody (Abcam, ab125011), Alix-antibody (CST, 92880), Lamp2b-antibody (Abcam, ab18529), anti-rabbit IgG, HRP-linked Antibody (CST, 7074) were used as the antibodies.

#### Autophagic flux analysis

The adenoviral vector carrying RFP-GFP-LC3 (HB-AP2100001) were purchased from HANBIO. This construct fluorescence depends on the difference in pH between the acidic autolysosome and the neutral autophagosome, and the exhibited red/green (yellow) or red fluorescence makes it possible to monitor progression of autophagic flux. To analyze the autophagic flux in rat chondrocytes, the cells were planted on cover slips that had been retained in 24-well plates were treated with: induced with *IL-1β*, or co-culture with MSCs^SC^-Exos for 48 h, or MHY1485, up to the need of the trial. Subsequently the cells were infected with the RFP-GFP-LC3 adenovirus for 24 h. Finally cultured cells on the cover slips were washed in cool PBS and fixed in 4% PFA, stained the nuclear with DAPI for immunostaining and detected for the images using a laser scanning confocal microscope (Leica, TSC SP8). For each condition, at least 4 RFP-GFP-LC3-transfected images were subjected to fluorescence analysis, and the percentage of transfected cells showing puncta RFP-GFP-LC3 were used to indicate the accumulation of autophagosomes.

#### MiR-199-3p loading by electroporation

The miR-199-3p mimic was loaded inside exosomes derived MSCs^SC^ by electroporation. Briefly, exosomes at a total protein concentration of 10 μg and 500 nmol miR-199-3p mimic were mixed in 400 μl of electroporation buffer. After electroporation at 250 V and 125 μF in a 4 mm electroporation cuvette using a Gene Pulser Xcell^™^ system (Bio-Rad Laboratories, CA), the mixture was incubated at 37 °C for 30 min to ensure the recovery of the exosome membrane. The exosomes were ultracentrifugated for 70 min at 100,000 ×*g* and 4 °C to remove the unloaded miR-199-3p. Then the pellets were resuspended in PBS, and the generated exosomes were stored at − 80 °C or used instantly. The Cy3-labeled miR-199-3p was used to measure the loading efficiency of miR-199-3p, which was quantified using a fluorimeter with excitation at 532 nm and emission at 580 nm (SpectraMax M5, Molecular Devices, USA). The loading efficiency of miR-199-3p was determined by calculating the encapsulated miR-199-3p over total initial miR-199-3p.

#### RNA extraction, reverse transcription, and quantitative real-time (RT)-polymerase chain reaction (PCR)

Total RNA was isolated from tissues or cultured cells using Trizol reagent (Invitrogen, 15596-026). HiScript^®^ III 1st Strand cDNA Synthesis Kit was used to synthesize the first-strand cDNA (Vazyme, R312-02, China). Real-Time PCR was used on a light cycler (Roche, Basel, Switzerland) to evaluate the mRNAs expression using ChamQ^™^ Universal SYBR^®^ qPCR Master Mix (Vazyme, Q711-03) and Actin as an endogenous control. Furthermore, miRNA-specific real-time qPCR was performed in accordance with the manufacturer instructions (Vazyme, Q711-03), and U6 small nuclear RNA (snRNA) was employed as a control to determine miRNAs. Primer sequences are shown in Table 1 (Additional file 1).

**Table 1:** The primer sets for qRT-PCR were listed below

Gene	Forward primer (5ʹ-3ʹ)	Reverse primer (5ʹ-3ʹ)
rno ACTIN	AAGGCCAACCGTGAAAAGAT	TGGTACGACCAGAGGCATA
rno MAP1LC3B	TGTTAAGCCCCTACCAAGGC	GCATGGCACTCAGTTTCTGG
ATG9A	CGAGGCTGGTAACTGGAATC	CCCAGCCCTTAGCACTTAGAC
Atg4C	GTAGTCCGAGACCGCAGGAG	GAGCCTAGCGCCGTGTATTT
TGFB1	GACTCTCCACCTGCAAGACC	GGACTGGCGAGCCTTAGTTT
WIPI2	GCATGGAGACGACCAGTGAA	TTGGCGTCCTGTCCTTTACC
HTT	TGCCCGTGTAAAGTGTGTGA	AAGTGCCATAGGGTGTTGGG
PIK3C3	AGATGGTCGAAGGGATGGGT	AGGGCGTGAACACTTTCGTC
AMBRA1	GGCGTTCGTGTGTTCGATG	CTCATGGAAATCTGCGAGGC
TP53	CCCCTGAAGACTGGATAACTGT	CAGGAGCTGACACTTGGAGG
BCL2L1	AGATTCGGCACGAGCAGTC	TGGGCTCAACCAGTCCATTG
BNIP3	TTGGGTGGGCAGTTGTGTTA	TACAGCATGTCAAGGAGGCA
MAPK8	CTCTCCAGCACCCGTACATC	TTGACAGACGGCGAAGAGAC
CLN3	CGCTAACTCCTAACACACGC	GTTTCCAGATGCCTGCTCCT
VTILA	CTCAGAGAACCAGAGGGCAC	CCCAGTCAGAATCCGAGAGC
ATP6V0A1	GAGGACGCAGAAGAGCCTAC	AGGACGCAGTGTTGGAGATG
IGF1	CAAAATGAGCGCACCTCCAA	CTTCAGCGGAGCACAGTACA
HDAC6	GCTCAGGAATCTTGGGAGCA	AAGAATCTTGGCCGGTGGAG
BAX	CACGTCTGCGGGGAGTCAC	TTCTTGGTGGATGCGTCCTG
BAK1	GTTTCTCGGGCTAAAAGGCG	CCAGATGTGGAATGGCCCAG
MAPK14	GGATATTTGGTCCGTGGGCT	TCATGGCTTGGCATCCTGTTA
DAPK1	GGTTCCCACTCACTCCTTCTG	CCAAGTGACCGAAGTGAACC
SLC7A5	GCGTGAAAACTGCTGGCTAC	TCACCCCAACACAAGGGAAC
mTOR	CTGCACTTGTTGTTGCCTCC	TGGTCTAGCGTGCGAACAAT
hsa U6	CTCGCTTCGGCAGCACA	AACGCTTCACGAATTTGCGT
Rno/mmu U6	CTCGCTTCGGCAGCACA	AACGCTTCACGAATTTGCAT
rno/has/mmu miR-199a-3p	CGGCGGACAGTAGTCTGCACA	CAGTGCAGGGTCCGAGGTAT
rno/has/mmu miR-199a-3p stem loop primer	GTCGTATCCAGTGCAGGGTCCGAGGTATTCGCACTGGATACGACTAACCA	

#### Dual-luciferase reporter assay

The bioinformatics web software miRDB was employed to predict target genes and verify if miR-199a-3p and mTOR have binding sites. The wild-type mTOR dual-luciferase reporter vector (WT mTOR) and mutant mTOR dual-luciferase reporter vector (MUT mTOR) was constructed respectively, and then co-transfected into C28/I2 cells with miR-199a-3p mimic and the negative control. A miRNA control was employed as a negative control. To determine whether miR-199a-3p contributes to the effect of Exos^SC^ on OA model, C28/I2 cells were pretreated with Exos^SC^ for 24 h, then were transfected with the antagomir-199a-3p and the reporter plasmids. The activity of luciferase in C28/I2 cells was measured 48 h after transfection, and reporter tests were carried out according to the manufacturer’s instructions of a dual luciferase activity detection kit (Promega, E1910, USA). The activity of renilla luciferase was normalised to that of firefly luciferase and represrnted as % of the control.

### Sequencing of MSCs^SC^-Exos derived MiRNA

Total RNAs of MSCs^SC^-Exos were isolated and used for miRNA sequencing. The miRNA Library was built and sequenced at LC Bio Technology CO.,Ltd (Hangzhou, China), and was collected by the Illumina HiSeq 2500 platform.

### Statistical analysis

All experiments were conducted in duplicate or triplicate and were monitored by separate observers. Student’s *t*-test was employed to compare two groups, whereas the one-way analysis of variance (ANOVA) and Tukey’s multiple comparison test were used to compare three groups. GraphPad Prism 8.0 was used for all statistical analyses (GraphPad Software Inc., La Jolla, CA, USA). The data are shown as the mean ± standard error (SEM), and *p* < 0.05 regarded statistically significant. *p < 0.05, **p < 0.01, ***p < 0.001, ****p < 0.001, ns not significant.

## Results

### Isolation and identification of MSCs^SC^ and MSCs^SC^ derived exosomes

**Fig. 1:**
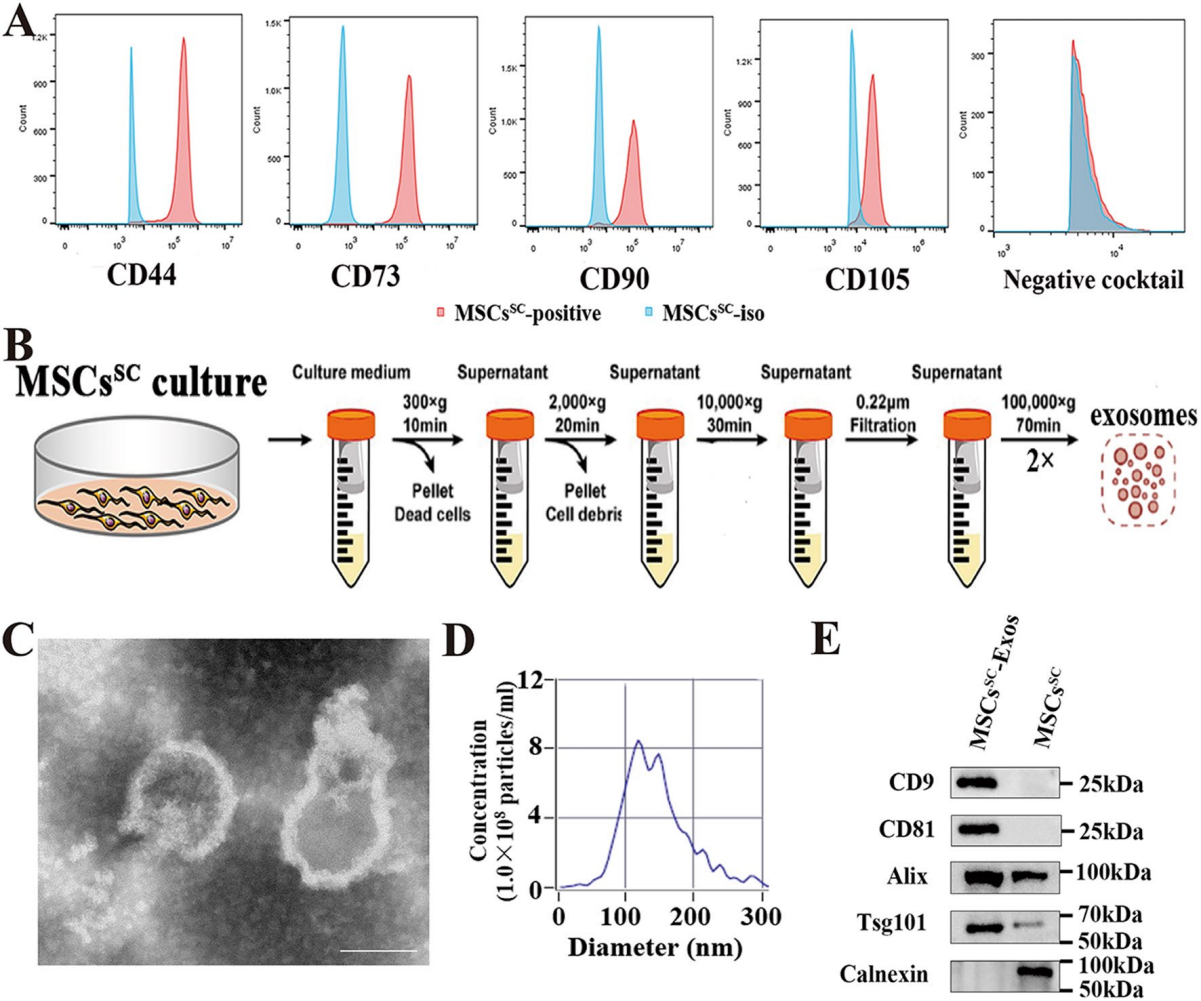
Identification of MSCs^SC^ and MSCs^SC^-Exos. **A** Flow cytometry analysis of MSCs^SC^ markers in P4-ADSCs, the solid red curves represent measured surface markers and the solid blue curves represent isotype controls. **B** Schematic illustration of MSCs^SC^-Exos isolation using differential centrifugation. **C** Transmission electron microscopy (TEM) analysis for the morphology of MSCs^SC^-Exos (Scale bar: 100 nm). **D** Nanoparticle tracking analysis (NTA) for measuring the size distribution and concentration of MSCs^SC^-Exos. **E** Western blot analysis of protein markers CD9, CD81, Alix, Tsg101, and Calnexin in MSCs^SC^-Exos and MSCs^SC^

The isolated human SC-derived MSCs were cultured as previous report [22], and then characterized by flow cytometry for the mesenchymal stem cell markers. The MSCs^SC^ are highly positive for the mesenchymal stem cells markers (CD44, CD73, CD105, CD90) and negative for CD34, CD11b, CD19, CD45, HLA-DR (human MSC negative cocktail) (Fig. 1A). Besides, the multipotency of isolated MSCs^SC^ with tri-lineage differentiation assay, including adipogenesis, chondrogenesis, and osteogenesis, was observed in our previous studies [2, 21]. The above research showed qualified MSCs^SC^ were generated. Next, MSCs^SC^-Exos were separately as previously described [42]. MSCs^SC^-Exos were isolated using differential ultracentrifuge method (Fig. 1B). The isolated MSCs^SC^-EVs displayed a sphere-shaped morphology and a size around 100 nm with Transmission electron microscopy (TEM) analysis (Fig. 1C). Nanoparticle Tracking Analysis (NTA) showed that the size distribution of most MSCs^SC^-EVs varied from 70 to 150 nm and the main peak diameter was 118.9 nm, while the particle concentration of MSCs^SC^-EVs was about 1.6 × 10^10^ particles/ml (Fig. 1D). Furthermore, the MSCs^SC^-EVs highly expressed CD9, CD81, Alix and Tsg101, but were negative for the endoplasmic reticulum membrane-related marker Calnexin, which only expressed in MSCs^SC^ cells (Fig. 1E). Taken together, these results demonstrate that the separated MSCs^SC^-EVs, which could meet the requirements of exosomal morphology, concentration, size distribution and protein markers, was verified to be highly quantified MSCs^SC^-Exos.

### MSCs^SC^-Exos repair the damaged articular cartilage in OA rat model

**Fig. 2:**
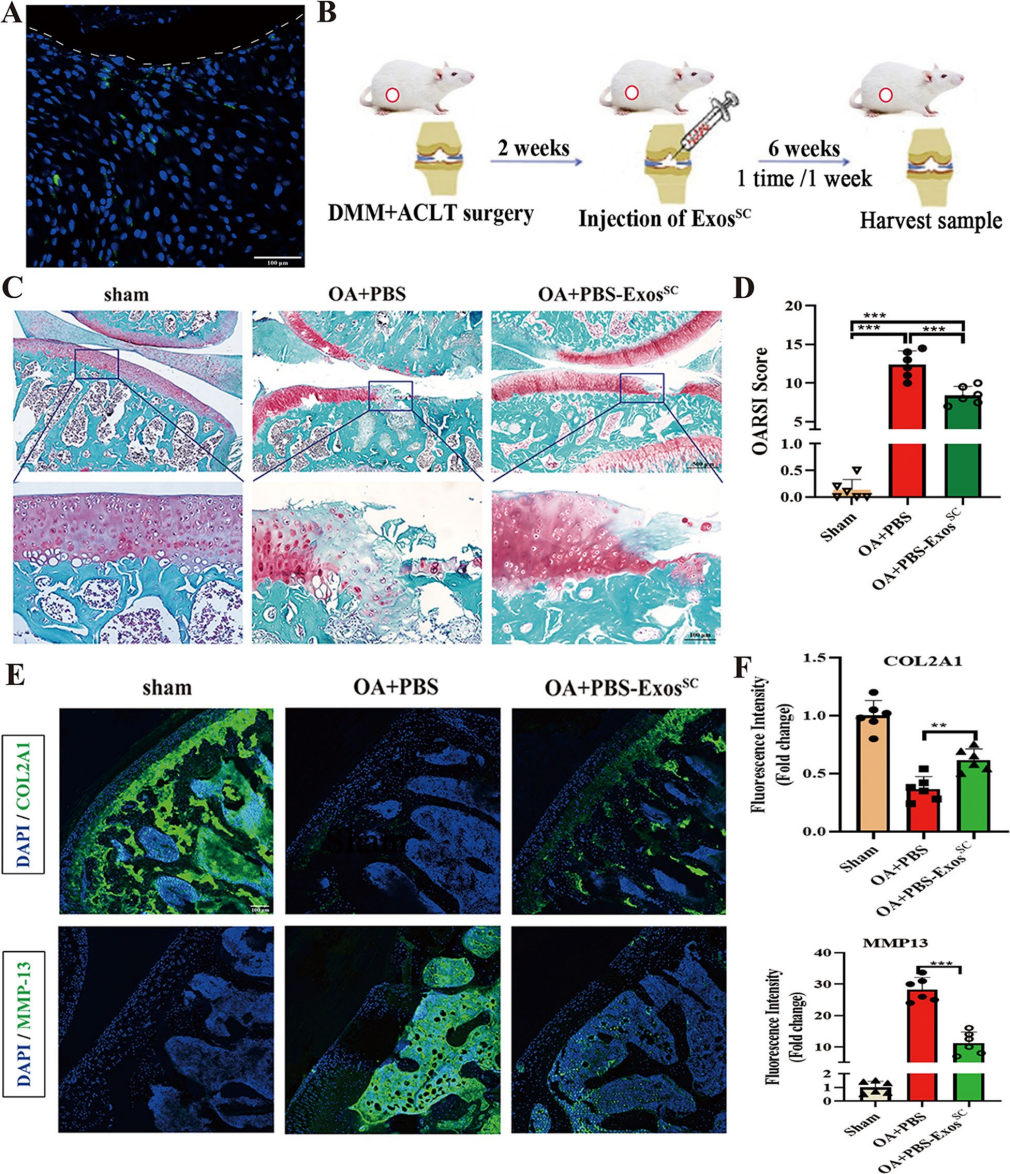
MSCs^SC^-Exos treatment repairs the damaged articular cartilage in OA rat model. **A** Representative image of DiO-labelled MSCs^SC^-Exos were uptaken by the damaged cartilage. White dotted lines indicate articular surface and the green dots indicate DiO-labelled MSCs^SC^-Exos. Scale bar: 100 μm. **B** Schematic diagram of MSCs^SC^-Exos treatment strategy in OA rats. **C** Safranin O/fast green staining of cartilage morphology in each group rats, scale bar: 500 μm (up) and 100 μm (down). **D** The OARSI scoring system quantify the severity of cartilage destruction. n = 6 for each group. **E** and **F** The immunofluorescence analysis of COL2A1 and MMP-13 in articular cartilage of OA rat model. n = 6 for each group. Scale bar: 100 μm. * p < 0.05, ** p < 0.01, *** p < 0.001, **** p < 0.001, ns not significant

To investigate the potential role of MSCs^SC^-Exos treatments in articular cartilage of OA rats, an experimental model of OA was induced in 8-week-old male SD rats by DMM + ACLT, a model that was previously established [40]. Firstly, to examine whether the MSCs^SC^-Exos could enter the damaged articular cartilage, the MSCs^SC^-Exos was labeled using Dio-staining. After 24h of intra-articular injection, the articular cartilage was isolated. The green fluorescent signal of Dio-labeled MSCs^SC^-Exos was seen in the area of destructive cartilage under the fluorescent microscope (Fig. 2A). Secondly, MSCs^SC^-Exos that diluted in PBS (PBS-Exos^SC^) or its negative control (PBS) were intra-articular injected on OA rats (Fig. 2B). The histomorphological change of articular cartilage in OA rats were examined with the Safranin O/fast green staining. The results showed that accelerated destruction of cartilage and loss of proteoglycan in the tibial cartilage were showed on the OA rats comparing to the sham rats, and obvious repair of cartilage and increase of proteoglycan were seen in the tibial cartilage of the exosome-treated group (PBS-Exos^SC^), compared to the PBS injected group (PBS) in OA rats (Fig. 2C). Accordingly, the Osteoarthritis Research Society International (OARSI) scoring including the summed scores of the tibia also revealed that the injection of MSCs^SC^-Exos significantly alleviated the cartilage erosion in these Exos^SC^-treated group comparing to only PBS-treated group (Fig. 2D). Then the change of OA-related protein levels in articular cartilage of OA rats was examined using the immunofluorescence experiment. Exos^SC^ treatment could dramatically reverse the OA-induced decrease of COL2A1, while down-regulate the increased expression of MMP13 in OA model (Fig. 2E, F). Briefly, these results revealed that MSCs^SC^-Exos could enter the damaged area of articular cartilage, promote cartilage repair and ECM synthesis and repair the cartilage destruction of OA rats.

### MSCs^SC^-Exos treatments prevent cartilage destruction by mediating the autophagy of OA rats

**Fig. 3:**
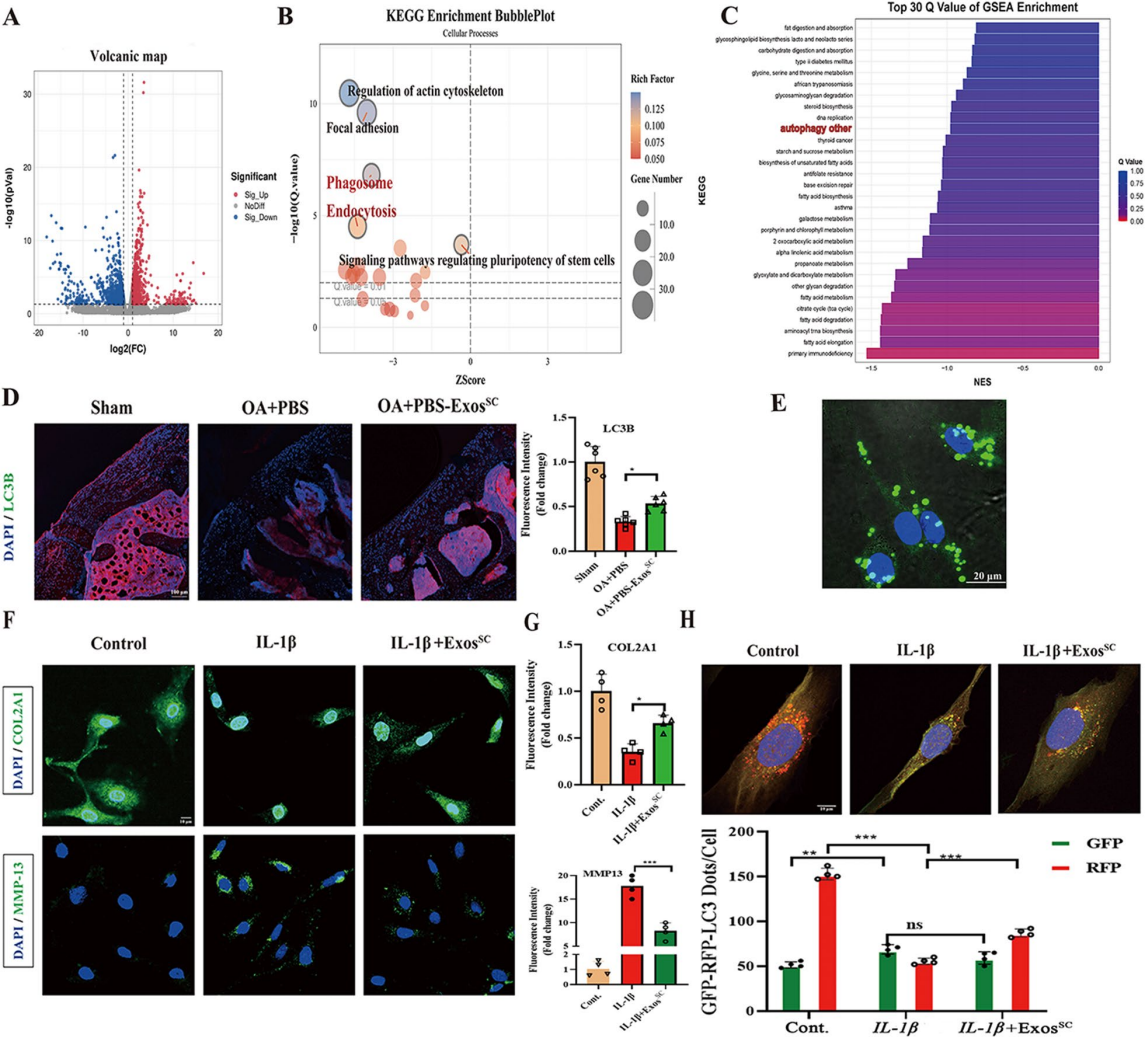
The autophagy level was up-regulated in OA rats treated with MSCs^SC^-Exos. **A** RNA-Seq analysis was conducted in total cartilage mRNA of OA rats, which was extracted from control group (PBS-treated, n = 2) and MSCs^SC^-Exos group (Exos^SC^-treated, n = 2). Volcanic map of different expression genes (DEGs) between control group and Exos^SC^ group. Red spots represent up-regulated genes and blue spots represent down-regulated genes. **B** KEGG enrichment for control group and Exos^SC^ group. **C** GSEA enrichment analysis of control group and Exos^SC^ group. **D** The immunofluorescence analysis of LC3B in articular cartilage of OA rat model. n = 6 for each group. scale bar: 100 μm. **E** Representative image of DiO-labelled Exos^SC^ absorbed by rat chondrocytes. Scale bar: 20 μm. **F** and **G** The immunofluorescence analysis of COL2A1 and MMP-13 in chondrocytes of OA model. n = 4 for each group. Scale bar: 10 μm. **H** Rat chondrocytes were infected with RFP-GFP-LC3 adenovirus and effects of Exos^SC^ on RFP-GFP- LC3 puncta. The numbers of RFP- and GFP-LC3 dots per cell were counted. n = 4 for each group. Scale bar: 10 μm. * p < 0.05, ** p < 0.01, *** p < 0.001, **** p < 0.001, ns not significant

To clarify the mechanism by which MSCs^SC^-Exos alleviated the cartilage destruction, RNA-seq was applied to analyze the gene expression of cartilage samples in DMM + ACLT-induced OA controls and MSCs^SC^-Exos treated OA rats. Heatmap showed a variety of identified 1065 down-regulated genes (*p* < 0.05) and 911 up-regulated genes (*p* < 0.05) in OA rats with MSCs^SC^-Exos therapy (Fig. 3A). According to the differentially expressed genes, KEGG enrichment was carried out. The data suggested that cellular process including phagosome process and endocytosis, were remarkably regulated, which are important autophagy-related biological process (Fig. 3B). Besides, gene set enrichment analysis (GSEA) was adopted to deeply analyze the RNA-seq data. Interestingly, the GSEA results revealed that MSCs^SC^-Exos treatment remarkably mediated autophagy process consistently (the top 10 of GSEA enrichment), which were highly correlated with our results that MSCs^SC^-Exos alleviate the cartilage destruction of OA rats (Fig. 3C).

To confirm the above-analyzed results of RNA-seq data, we investigated that whether the effect of MSCs^SC^-Exos on cartilage was related with autophagy process. First, the protein expression levels of LC3B, the critical components of autophagosomes [43], were detected on cartilage of OA rats with immunofluorescence assay. As shown in Fig. 3D, MSCs^SC^-Exos treatment significantly increased the expression level of LC3B in cartilage compared to the treatment of PBS on OA rats, suggesting an enhanced level of autophagy with MSCs^SC^-Exos therapy.

Second, to investigate the effect of MSCs^SC^-Exos was related with autophagy process in vitro, the *IL-1β-*induced OA chondrocyte model were generated. The protein levels of anabolism-related gene (COL2A1) and catabolism-related gene (MMP13) were determined. After *IL-1β* (10 ng/ml) induction for 24 h the protein levels of COL2A1 expression significantly reduced, while the protein levels of MMP13 highly up-regulated, indicating *IL-1β* could successfully imitate the OA microenvironment in vitro (Fig. 3F and G). Additionally, to confirm the MSCs^SC^-Exos could enter into chondrocytes, we incubated the primary rat chondrocyte with DiO-labelled MSCs^SC^-Exos for 12h, and then observed the green fluorescence signals of DiO in chondrocyte. The result showed that the primary rat chondrocytes could largely uptake the MSCs^SC^-Exos, which mainly distributed in the cytoplasm of chondrocytes (Fig. 3E). Accordingly, to estimate the effect of MSCs^SC^-Exos on chondrocyte function in vitro, we treated chondrocytes with MSCs^SC^-Exos (1 × 10^10^ exosome particles/ml) in the presence or absence of *IL-1β*. The immunofluorescence results showed MSCs^SC^-Exos addition significantly reversed the effect of *IL-1β* on COL2A1 and MMP13 of rat chondrocytes (Fig. 3F and G). Subsequently, the effect of MSCs^SC^-Exos on autophagy process of rat chondrocytes was evaluated in vitro. The rat chondrocytes infected with the RFP-GFP-LC3 adenovirus, a specific marker of autophagosome formation that relies on the differences in GFP and RFP fluorescence under acidic conditions, were to be monitored the progression of autophagic flux. Colocalization of GFP and RFP signals (yellow dots) indicates a lack of phagophore fusion or of autophagosome and lysosome fusion, whereas RFP only signals (red dots) indicate the presence of autolysosomes. To enhance the levels of autophagy, the rat chondrocytes were precultured with condition medium of 4% FBS for 24 h. The results of Fig. 3H showed that MSCs^SC^-Exos treatment significantly increased the number of red/yellow dots on rat chondrocytes, while the number of red/yellow dots significantly decreased on rat chondrocytes with only *IL-1β* treatment, comparing to control chondrocytes. Collectively, these data suggested that the enhanced autophagy process with MSCs^SC^-Exos treatment might be a pivotal reason for the therapy of the destructed cartilage in OA model.

### MSCs^SC^-Exos up regulated the autophagy process via inhibition of mTOR expression

**Fig. 4:**
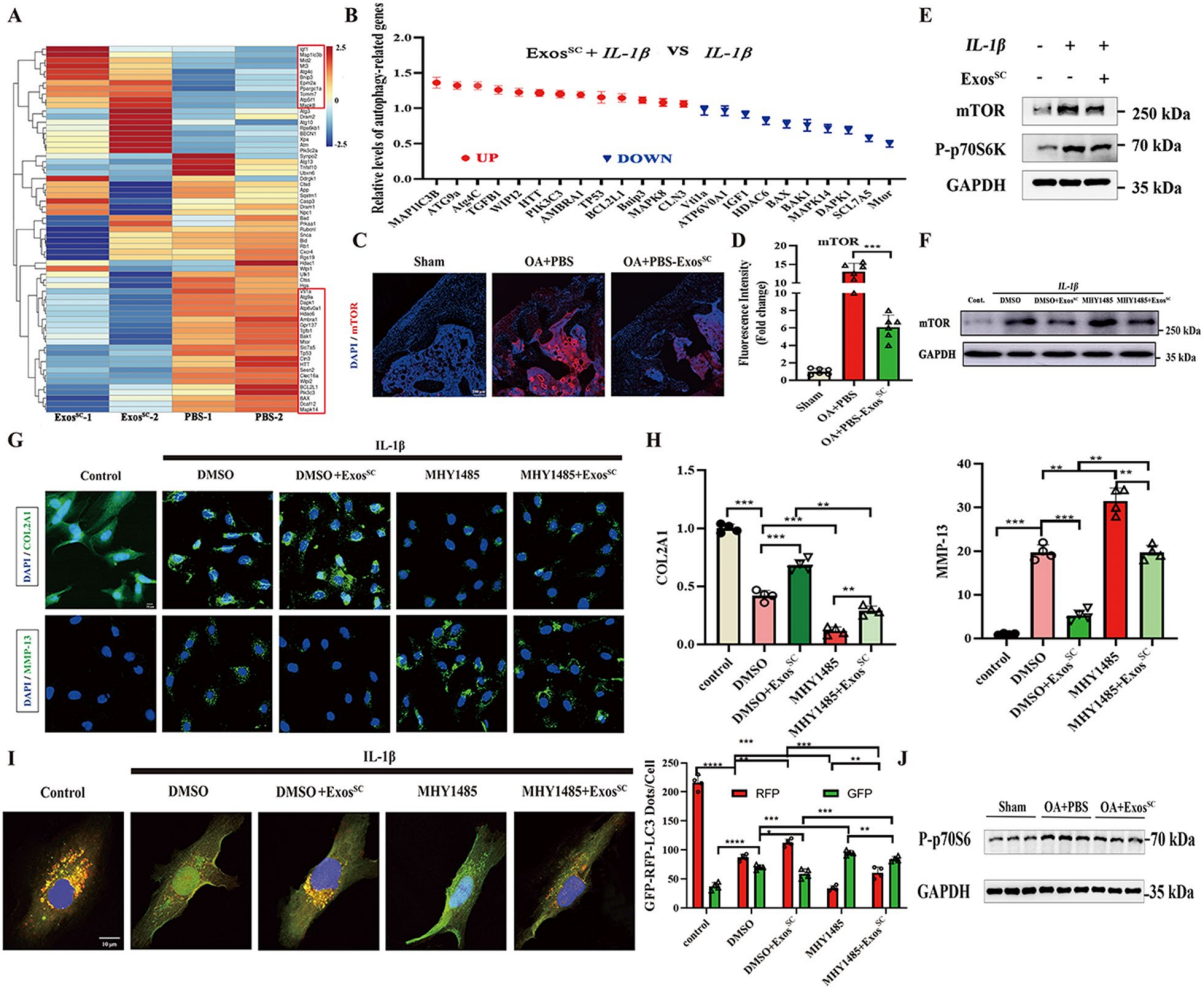
MSCs^SC^-Exos up regulated the autophagy process via inhibiting the mTOR expression. **A** Heatmap showing the hierarchical cluster of differential expression genes in OA cartilage treated with MSCs^SC^-Exos or PBS. **B** Relative expression levels of the autophagy-related genes in primary chondrocytes treated with *IL-1β* and MSCs^SC^-Exos by qRT-PCR. **C**, **D** The immunofluorescence analysis of mTOR in articular cartilage of OA rat model. n = 6 for each group. Scale bar: 100 μm. **E** Protein levels of mTOR and P-p70s6 were examined in chondrocytes treated with *IL-1β* and MSCs^SC^-Exos by western blotting. **F** Protein level of mTOR was examined by western blotting in chondrocytes treated with *IL-1β*, MSCs^SC^-Exos and MHY1485. **G** and **H** The immunofluorescence analysis of COL2A1 and MMP-13 in chondrocytes of OA chondrocytes treated with MHY1485. n = 4 for each group. Scale bar: 10 μm. **I** Rat chondrocytes were infected with RFP-GFP-LC3 adenovirus and effects of MHY1485 on RFP-GFP- LC3 puncta. The numbers of RFP- and GFP-LC3 dots per cell were counted. n = 4 for each group. Scale bar: 10 μm. **J** Protein levels of P-p70s6 were examined in articular cartilage in OA rat model by western blotting. * p < 0.05, ** p < 0.01, *** p < 0.001, **** p < 0.001, ns not significant

To excavate molecular mechanisms of MSCs^SC^-Exos up-regulated the autophagy of OA cartilage, the autophagy-related genes of RNA-seq data on OA cartilage between Exos^SC^ group and control group were analyzed. In order to explore more autophagy-related genes between Exos^SC^ group and control group, we choose two groups from the above-detected RNA-seq data, and the results showed that a variety of identified 22 down-regulated genes and 11 up-regulated genes in OA rats with Exos^SC^ therapy (Fig. 4A). Then we validated the mRNA levels of the above autophagy-related genes in *IL-1β*-treated chondrocytes using RT-PCR. The RT-PCR results showed that 23 genes were differentially expressed with MSCs^SC^-Exos therapy on *IL-1β*-treated chondrocytes (Fig. 4B). Among these differentially expressed genes, the change of mTOR mRNA was the most obvious among these genes (Fig. 4B). As known, mTOR was an important negative regulator on autophagy process [44], and mTOR inhibition up-regulated autophagy could promote the repair of damaged cartilage [45, 46], so we focused on the mTOR. Subsequently we validated the protein levels of mTOR in OA rat cartilage using immunofluorescence assay. The results demonstrated that mTOR protein level of cartilage on Exos^SC^-treated OA group was significantly down-regulated compared with control group (Fig. 4C and D). Similar to the in vivo results, the in vitro experiment showed MSCs^SC^-Exos treatment could dramatically reverse the *IL-1β*-induced increased the expression of mTOR (Fig. 4E).

To further confirm mTOR plays a crucial role in MSCs^SC^-Exos up-regulated the autophagy of OA cartilage, MHY1485 (an mTOR activator) was added in the *IL-1β-*induced OA rat chondrocyte models before 36h stimulation of MSCs^SC^-Exos. MHY1485 at 50 μmol/l concentration for 12h could effectively enhance the mTOR expression without significant influence on cell viability, according to the dose–response experiments (Additional file 1: Fig. S1). MHY1485 treatment increased the protein levels of mTOR, while MSCs^SC^-Exos treatment could reverse the increased protein levels of mTOR in the rat chondrocytes (Fig. 4F). The results of anabolism-related gene (COL2A1) and catabolism-related gene (MMP13) showed that MSCs^SC^-Exos treatment could reverse the decreased protein levels of COL2A1 and the increased protein levels of MMP13 due to the addition of MHY1485 in the OA rat chondrocytes (Fig. 4G, H). Then, the effect of MHY1485 and MSCs^SC^-Exos on autophagy process of rat chondrocytes was evaluated in vitro. To enhance the levels of autophagy, the rat chondrocytes were precultured with condition medium of 4% FBS for 24 h. The results of Fig. 4I showed that MHY1485 treatment significantly decreased the number of red/yellow dots on rat chondrocytes, while MSCs^SC^-Exos addition significantly reversed the effect of MHY1485 on the autophagy process of OA rat chondrocytes.

Furthermore, the mTOR signal pathway of MSCs^SC^-Exos therapy was deeply investigated in vivo and in vitro. The expression levels of p-p70s6, the downstream of the mTOR signal pathway, were significantly decreased on OA cartilage (Fig. 4E) and *IL-1β*-mediated chondrocytes (Fig. 4J) with MSCs^SC^-Exos therapy. Above all, these results revealed that MSCs^SC^-Exos enhanced the levels of autophagy in OA cartilage and chondrocyte through the inhibition of mTOR signal pathway, to protect the cartilage in OA microenvironment.

### miR-199a-3p transferred by MSCs^SC^-Exos decrease the expression of mTOR in OA model

**Fig. 5:**
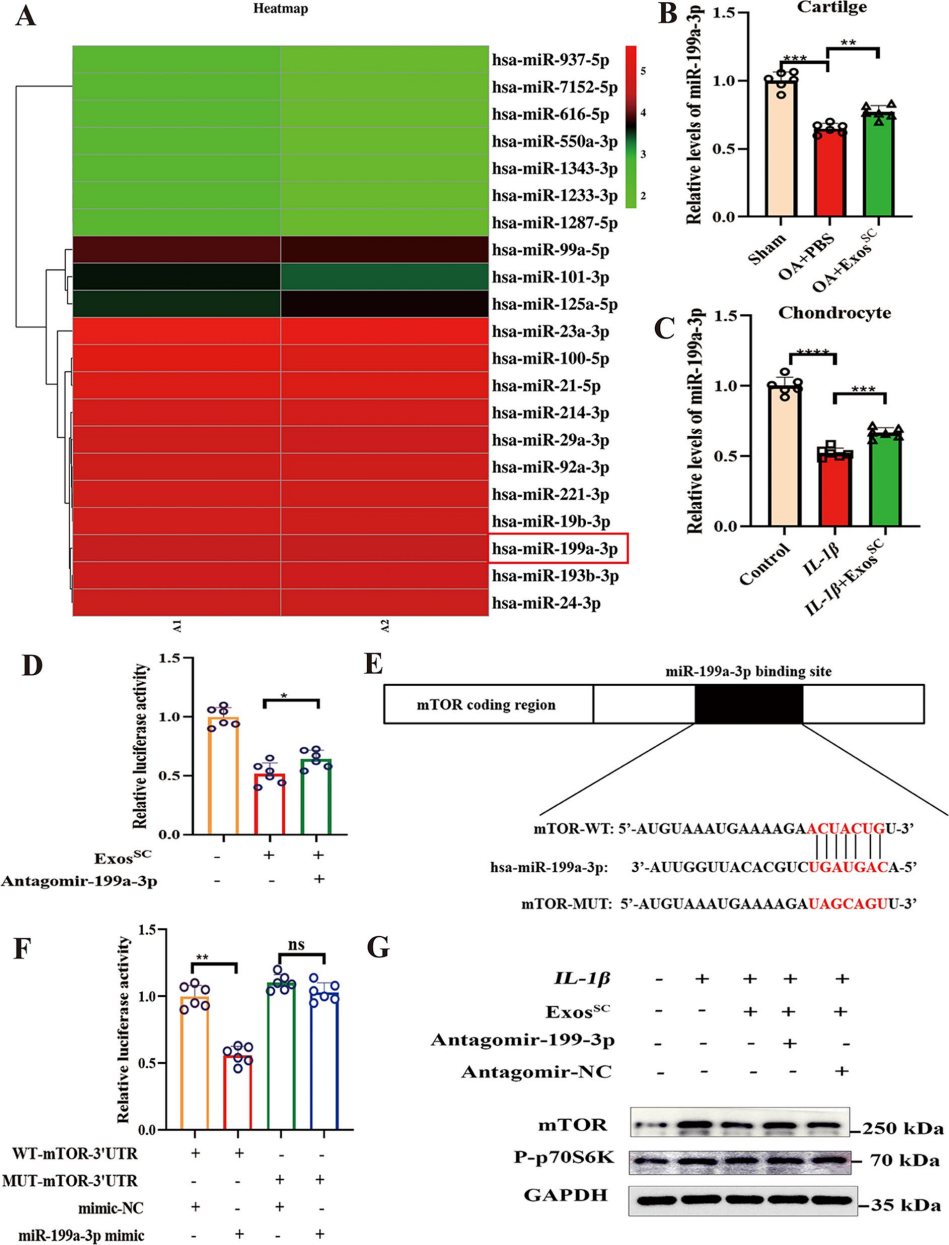
miR-199a-3p transferred by MSCs^SC^-Exos decrease the expression of mTOR in OA model. **A** Heatmap showing the hierarchical cluster of differential miRNA expression in MSCs^SC^-Exos. **B** The relative expression of miR-199a-3p on OA cartilage with MSCs^SC^-Exos treatment was detected by RT-PCR. **C** The relative expression of miR-199a-3p on chondrocytes with MSCs^SC^-Exos treatment was detected by qRT-PCR. **D** The effects of MSCs^SC^-Exos and antagomir-199a-3p on the luciferase activity of the *IL-1β*-induced chondrocytes. **E** Schematic representation of a predicted binding site of miR-199a-3p in the 3ʹUTR of mTOR mRNA, and the mutant mTOR 3ʹUTR. **F** The luciferase activity was determined using the dual-luciferase reporter system. **G** Protein levels of mTOR and P-p70s6 were examined by western blotting. * p < 0.05, ** p < 0.01, *** p < 0.001, **** p < 0.001, ns not significant

The underlying mechanism by which MSCs^SC^-Exos decreased mTOR in OA cartilage were explored. As miRNAs are the important functional cargos in exosomes, we deduced that the miRNAs in MSCs^SC^-Exos contributed to affect mTOR expression on cartilage. The miRNA expression profiles of MSCs^SC^-Exos were investigated by using highthroughput sequencing (miRNA-seq) (Fig. 5A). Among the most abundant miRNAs in MSCs^SC^-Exos, mir-199a-3p attracted our attention: First, it was one of most highly enriched miRNAs in MSCs^SC^-Exos, also reported by Ragni et al. 2020 [47]. Second, previous researched reported that miR-199a-3p was crucial in maintaining normal function of chondrocytes, including: facilitating chondrocyte proliferation, suppressing chondrocyte apoptosis and playing an anti-catabolic role in OA cartilage [48, 49]; and the expression of miR-199a-3p in chondrocyte was highly decreased in OA progression [48, 50, 51]. Third, it is predicted to be the potential microRNAs that target the 3’UTR of mTOR mRNA with online bioinformatics methods, and were verified on endothelial cells, hepatocellular carcinoma [47, 52]. Therefore, we speculated that mir-199a-3p might play a role in MSCs^SC^-Exos-mediated expression of mTOR in OA cartilage. Then the levels of miR-199a-3p in OA cartilage with MSCs^SC^-Exos therapy were determined. The result showed that the level of miR-199a-3p in cartilage of OA rats was significantly upregulated after MSCs^SC^-Exos treatment (Fig. 5B). Consistently, in vitro trial showed that level of miR-199a-3p in rat chondrocytes cultured with MSCs^SC^-Exos was much higher compared to rat chondrocytes cultured *IL-1β* alone (Fig. 5C).

Furthermore, we further detected whether miR-199a-3p contribute to the effect of MSCs^SC^-Exos on OA model using the luciferase reporter assay. The result showed that antagomir-199a-3p the significant reduction of the luciferase activity with MSCs^SC^-Exos addition (Fig. 5D). Subsequently, we identified considerable complementarity between the seed region of the miR-199a-3p and the 3’UTR of mTOR (Fig. 5E). Notably, a distinct decrease (p < 0.01) in luciferase activity was measured in C28/I2 cells co-transfected with miR-199a-3p mimic and pmiR-mTOR (WT). In addition, a mutation of the miR-199a-3p binding sequence located at 16-22nt of the mTOR 3’UTR (Mut) evidently abrogated the repression of the luciferase activity owing to miR-199a-3p overexpression (Fig. 5F). Finally, the protein expression of mTOR were determined with MSCs^SC^-Exos and antagomir-199a-3p addition in vitro. A distinct reduction of mTOR protein was observed in chondrocytes cultured with MSCs^SC^-Exos, while antagomir-199a-3p reversed the decrease of mTOR with OA progression (Fig. 5G). In addition, the protein expression of P-p70s6, the downstream of the mTOR signal pathway, presented the similar change in chondrocytes. (Fig. 5G). Above all, these results indicated that the MSCs^SC^-Exos downregulated the mTOR expression levels and the mTOR signal pathway, highly dependent on the exosomal miR-199a-3p.

### Antagomir-199a-3p abrogates MSCs^SC^-Exos therapeutic effect on damaged cartilage

**Fig. 6:**
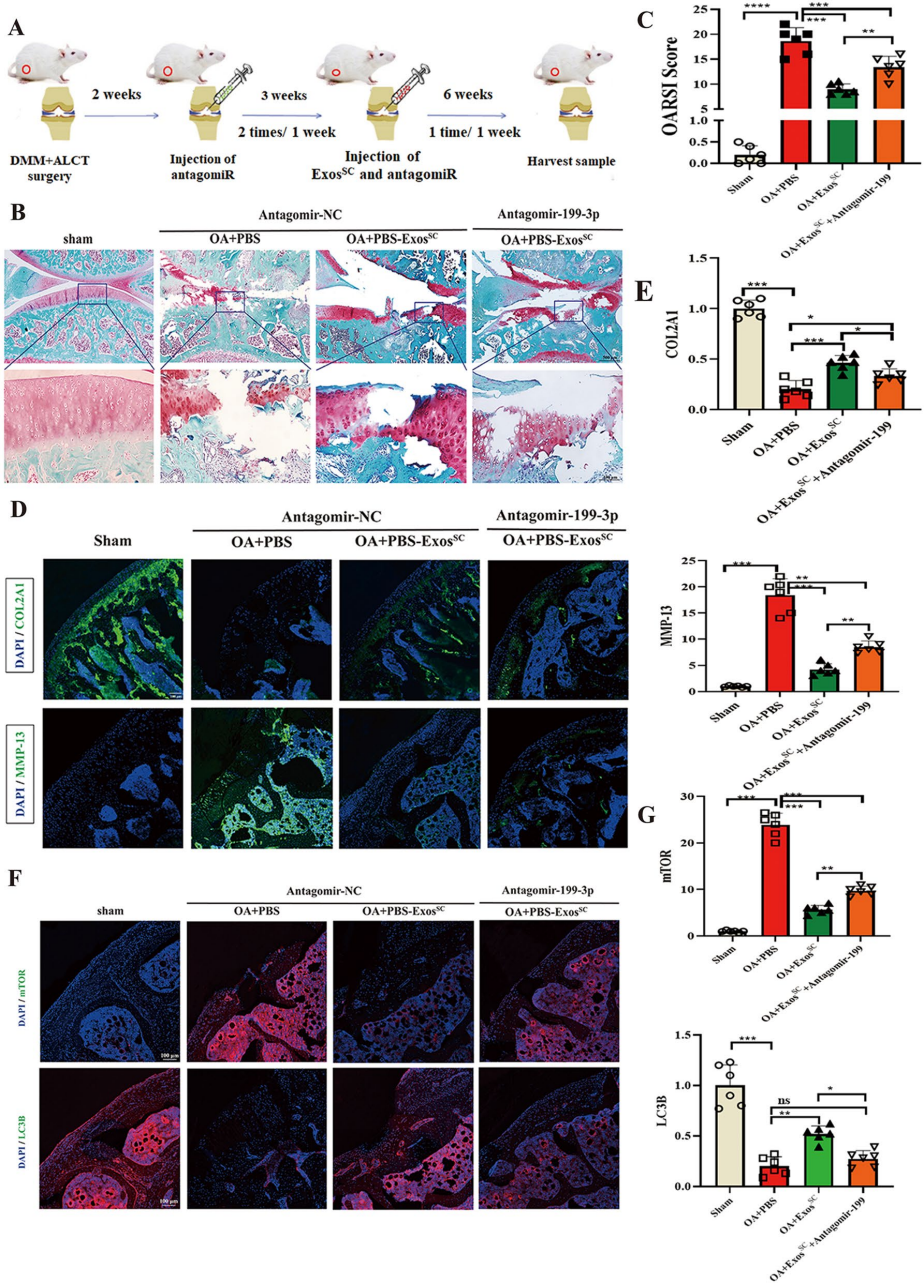
Antagomir-199a-3p abrogates MSCs^SC^-Exos therapeutic effect on damaged cartilage in OA model. **A** Schematic diagram of antagomiR-199a-3p and MSCs^SC^-Exos treatment strategy in OA rats. **B** Safranin O/fast green staining of cartilage morphology in each group rats, scale bar: 500 μm (up) and 100 μm (down). **C** The OARSI scoring system quantify the severity of cartilage destruction. n = 6 for each group. **D** and **E** The immunofluorescence analysis of COL2A1 and MMP-13 in articular cartilage in OA rat model. n = 6 for each group. Scale bar: 100 μm. **F** and **G** The immunofluorescence analysis of mTOR and LC3B in articular cartilage in OA rat model. n = 6 for each group. Scale bar: 100 μm. * p < 0.05, ** p < 0.01, *** p < 0.001, **** p < 0.001, ns not significant

To further analyze the effect of miR-199a-3p on MSCs^SC^-Exos-mediated cartilage protection, antagomiR-199a-3p were injected into the articular cavity of the OA rats. Figure 6A showed the established strategies with the injection of MSCs^SC^-Exos and antagomiR-199a-3p. After 11 weeks of OA surgery, the knee joints was harvested and Safranin O & fast green staining were used for histological analysis. The results showed that antagomiR-199a-3p abolished the effect of MSCs^SC^-Exos-mediated cartilage protection in OA rats (Fig. 6B). The OARSI scoring results showed that the cartilage destruction was more severe in OA rats with antagomiR-199a-3p treatment, even though the therapeutic MSCs^SC^-Exos were intra-articular injected in all OA rats (Fig. 6C). Furthermore, the protein levels of anabolism-related gene (COL2A1) and catabolism-related gene (MMP13) was detected in OA cartilage. The results of immunofluorescence presented that antagomiR-199a-3p injection reversed the MSCs^SC^-Exos derived therapeutic effect, including the increase of COL2A1 and decrease of MMP13 (Fig. 6D, E). These above results demonstrated that intra-articular injection of antagomiR-199a-3p could be used to inhibit the therapeutic function of miR-199a-3p derived from MSCs^SC^-Exos in OA rats.

In the above sections, we revealed that MSCs^SC^-Exos enhanced the levels of autophagy in OA cartilage through downregulating the mTOR signal pathway, which is highly dependent on the exosomal miR-199a-3p. Then antagomiR-199a-3p were injected into the articular cavity of the OA rats to further vertify the mechanism of miR-199a-3p derived from MSCs^SC^-Exos in vivo. The immunofluorescence expriment was used to analyze the expression of related autophagy proteins in OA cartilage with antagomiR-199a-3p or MSCs^SC^-Exos treatment. The protein expression of LC3B and mTOR in OA cartilage were remarkably increased with antagomiR-199a-3p, upon the decreased effect of MSCs^SC^-Exos in vivo (Fig. 6F, G).

### Engineered MSCs^SC^-Exos mediated delivery of miR-199a-3p to chondrocyte in vitro

The above results showed that MSCs^SC^-Exos promoted the repair of injured cartilage via increasing the levels of autophagy in OA model, which is highly dependent on the exosomal miR-199a-3p. To exert the best effect of MSCs^SC^-Exos on the injured cartilage, we develop engineered MSCs^SC^-Exos to realize specific delivery of miR-199a-3p to chondrocytes in articular cartilage.

**Fig. 7:**
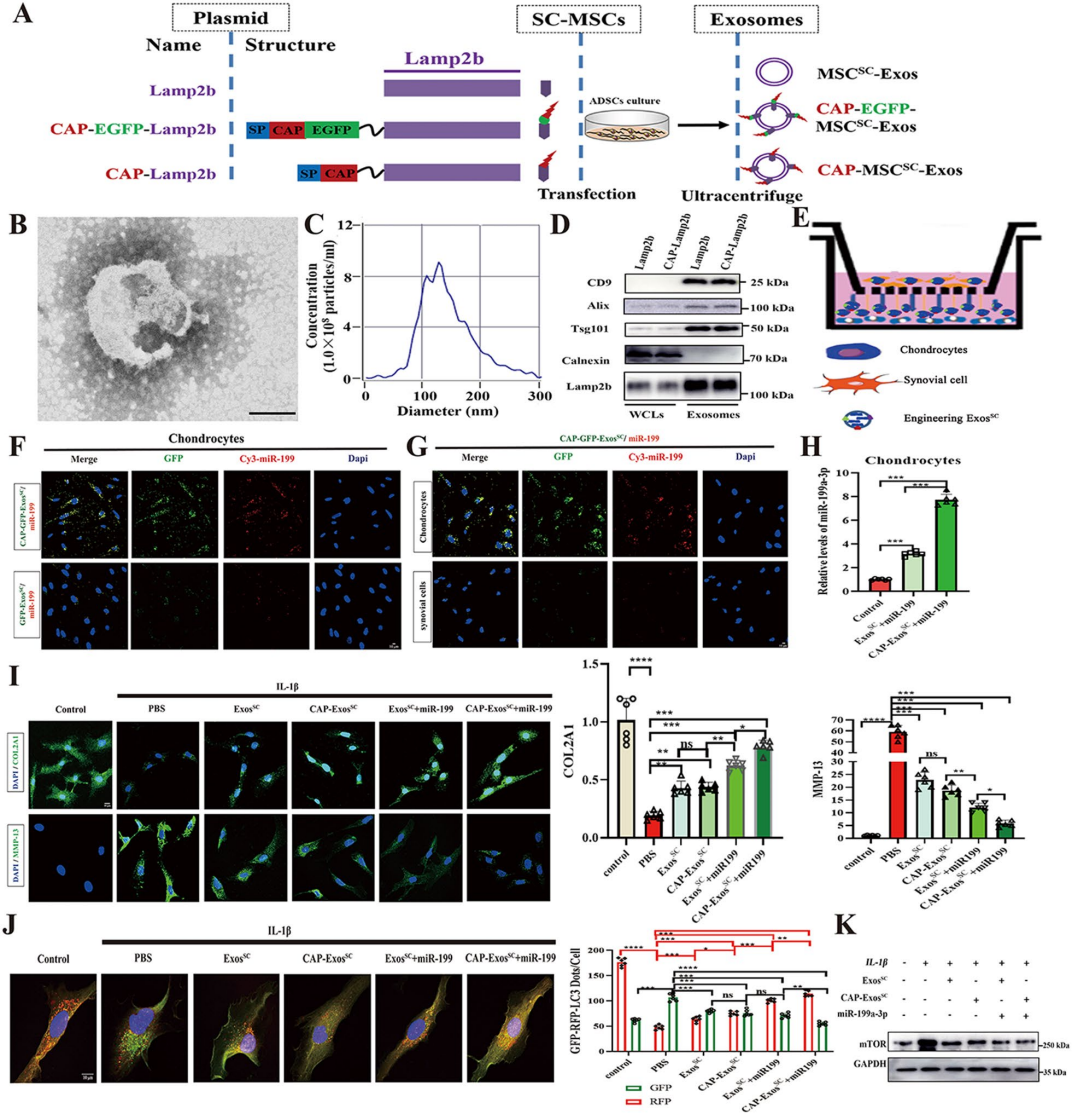
**A** Schematic diagram of the plasmid constructs containing Lamp2b, CAP-EGFP-Lamp2b and CAP-Lamp2b, and the engineering exosomes derived from MSCs^SC^. **B** Transmission electron microscopy (TEM) analysis for the morphology of CAP-MSCs^SC^-Exos (Scale bar: 100 nm). **C** Nanoparticle tracking analysis (NTA) for measuring the size distribution and concentration of CAP-MSCs^SC^-Exos. **D** Western blot analysis of protein markers CD9, CD81, Alix, Tsg101, and Calnexin in whole cell lysates (WCLs) and purified exosomes including CAP-MSCs^SC^-Exos and MSCs^SC^-Exos. **E** Schematic illustration of rat chondrocytes and synovial cells co-culture assays. **F** Efficient delivery of miR-199a-3p into chondrocytes by CAP-EGFP-MSCs^SC^-Exos. Scale bar: 10 μm. **G** Specific delivery to chondrocytes instead of synovial cells by CAP-EGFP-MSCs^SC^-Exos. Scale bar: 10 μm. **H** The relative expression of miR-199a-3p in chondrocytes treated with different MSCs^SC^-Exos delivery systems was detected by RT-PCR. **I** The immunofluorescence analysis of COL2A1 and MMP-13 in chondrocytes of OA model treated with different MSCs^SC^-Exos delivery systems. n = 6 for each group. Scale bar: 10 μm. **J** Rat chondrocytes were infected with RFP-GFP-LC3 adenovirus and effects of different MSCs^SC^-Exos delivery systems on RFP-GFP- LC3 puncta. The numbers of RFP- and GFP-LC3 dots per cell were counted. n = 6 for each group. Scale bar: 10 μm. **K** Protein level of mTOR in chondrocytes of OA model treated with different MSCs^SC^-Exos delivery systems was examined by western blotting. * p < 0.05, ** p < 0.01, *** p < 0.001, **** p < 0.001, ns not significant

To realize specific delivery of miR-199a-3p to chondrocytes for repairing the injured cartilage, we engineer the MSCs^SC^-Exos-based vehicle to realize the specific delivery to cartilage. The chondrocytes-affinity peptide (CAP) motif could effectively deliver miRNA to chondrocytes in the keen joint [39]. The plasmid CAP-Lamp2b (lysosomal associated membrane glycoprotein 2b) includes a glycosylation sequence (GNSTM), a CAP sequence (DWRVIIPPRPSA), and a glycine-serine spacer at the N-terminus of the Lamp2b protein was constructed, according to the previous reports [31, 37, 38]. Transfecting this plasmid to MSCs^SC^ allows the production of CAP-labeled exosomes (CAP-MSCs^SC^-Exos). Meanwhile, the other two plasmid encoding Lamp2b and CAP-EGFP-Lamp2b, were constructed respectively, according to the previous report [31]. Plasmid lamp2b serves as a control without a CAP sequence. Plasmid CAP-EGFP-lamp2b contains an EGFP protein after CAP to give fluorescently labeled CAP-EGFP-exosomes (CAP-EGFP-MSCs^SC^-Exos). After transfecting these plasmids to MSCs^SC^, MSCs^SC^-Exos were isolated using differential ultracentrifuge method [42] (Fig. 7A). Many previous researches have proved that there are no significant differences among the exosomes derived from the cells transfected with the plasmids, including: dendritic cells, iMSCs (derivate of induced pluripotent stem cells) [31, 37, 53, 54]. Therefore, the isolated CAP-MSCs^SC^-EVs was chosen to demonstrated the characters of engineering exosomes derived from MSCs^SC^. The isolated CAP-MSCs^SC^-EVs displayed a sphere-shaped morphology and a size around 120 nm with TEM analysis (Fig. 7B). NTA showed that the size distribution of most CAP-MSCs^SC^-EVs varied from 90 to 170 nm and the main peak diameter was 126.6 nm (Fig. 7C). Furthermore, Western blotting analysis of the protein lysates from the MSCs^SC^ and purified EVs showed that the CAP-MSCs^SC^-EVs and MSCs^SC^-EVs highly expressed CD9, Alix, Tsg101 and Lamp2b, but were negative for the endoplasmic reticulum membrane-related marker Calnexin, which only expressed in the corresponding transfected MSCs^SC^ (Fig. 7D). Taken together, these results demonstrated that the separated CAP-MSCs^SC^-EVs, which could meet the requirements of exosomal morphology, concentration, size distribution and protein markers, was verified to be highly quantified CAP-MSCs^SC^-Exos.

Next, miR-199a-3p was loaded into the above engineering exosomes derived from MSCs^SC^ using electroporation, as the technology has been verified not to change the endogenous miRNA profiles of the engineering exosomes [54]. To better examine the successful loading of miR-199a-3p into the CAP-MSCs^SC^-Exos, Cy3-labeled miR-199a-3p was using. By measuring the free miR-199a-3p left in the solution and comparing to the total input using a fluorometer (excitation at 550 nm and emission at 570 nm), we estimated that the miR-199a-3p loading efficiency of MSCs^SC^-Exos was around 40% (Additional file 1: Fig. S2). To better investigate whether CAP-MSCs^SC^-Exos could specifically enter chondrocytes in vitro, Cy3-labeled miR-199a-3p was loaded into CAP-GFP-MSCs^SC^-Exos or control GFP-MSCs^SC^-Exos through electroporation. The rat chondrocytes were added with miR-199a-3p-loaded MSCs^SC^-Exos, and were detected for the images after 4h incubation using a laser scanning confocal microscope. The significant Cy3 and GFP signals were monitored in chondrocytes added with the CAP-GFP-MSCs^SC^-Exos but less signals were seen with the GFP-exosome (Fig. 7F).

Next, we sought to measure whether the specifically delivered miR-199a-3p takes its effect on chondrocytes in vitro. Primary rat chondrocytes (the lower chambers) and rat synovial cells (the upper chambers) were co-culture in 6-well migration chambers. First of all, miR-199a-3p-loaded CAP-GFP-MSCs^SC^-Exos were added in the co-culture system. After 4h incubation, the Cy3 and GFP signals were highly enriched in the chondrocytes compared to the synovial cells (Fig. 7E, G), indicated that the CAP sequence effectively guides MSCs^SC^-Exos into the chondrocytes. Later, the co-culture system was treated with CAP-MSCs^SC^-Exos/miR-199a-3p or MSCs^SC^-Exos/miR-199a-3p for 48-72 h. The co-culture trials showed that CAP-MSCs^SC^-Exos/miR-199a-3p treatment was significantly increased the level of miR-199a-3p in rat chondrocytes, comparing to MSCs^SC^-Exos/miR-199a-3p; although miR-199a-3p level of both groups were decreased comparing to control group (Fig. 7H). The chondrogenic proteins (COL2A1) and MMP13 expression of rat chondrocytes were determined. The rat chondrocytes with CAP-MSCs^SC^-Exos/miR-199a-3p treatment showed higher chondrogenic proteins (COL2A1) level and lower MMP13 expression, than that with MSCs^SC^-Exos/miR-199a-3p treatment (Fig. 7I).

Then, the effect of CAP-MSCs^SC^-Exos/miR-199a-3p or MSCs^SC^-Exos/miR-199a-3p treatment on autophagy process of rat chondrocytes was evaluated in vitro. To enhance the levels of autophagy, the rat chondrocytes were precultured with condition medium of 4% FBS for 24 h. The results of Fig. 7J showed that the CAP-MSCs^SC^-Exos/miR-199a-3p added group showed the highest number of red/yellow dots on rat chondrocytes, compared to the other MSCs^SC^-Exos treated groups. Then, the protein expression mTOR in chondrocytes were detected, as we revealed the MSCs^SC^-Exos enhanced the levels of autophagy in OA cartilage through downregulating the mTOR signal pathway. The WB results showed that the protein expression of mTOR in OA chondrocytes were remarkably decreased in all the MSCs^SC^-Exos-treated groups comparing to the PBS group; especially the group added with CAP-MSCs^SC^-Exos/miR-199a-3p showed the most significant changes (Fig. 7K).

Taken together, engineering exosomes from MSCs^SC^ guided by the CAP signal peptides, markedly increased the delivery efficacy of exosomal miR-199a-3p to chondrocytes, and showed the enhanced autophagy of chondrocytes, thereby presented therapeutic potential in OA cell model.

### Cartilage-targeted miR-199a-3p delivery by engineered MSCs^SC^-Exos toward a cell-free OA therapy in vivo

**Fig. 8:**
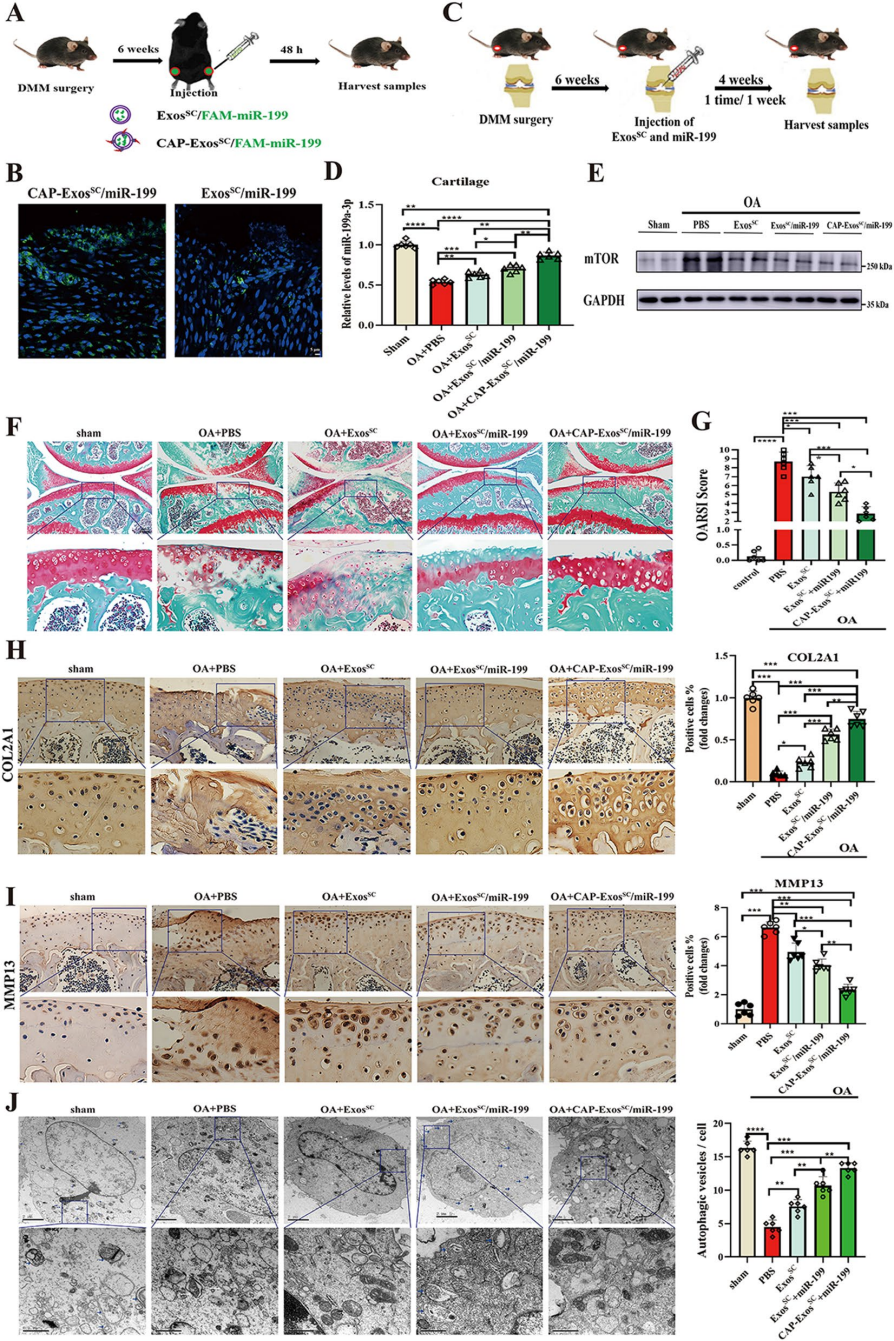
**A** Schematic illustration of the animal experimental procedure. MSCs^SC^-Exos or CAP-MSCs^SC^-Exos loading with FAM-miR-199a-3p were intra-articular injected into DMM-induced mice, and the joint samples were collected after 48 h. **B** Fluorescent images of cartilage tissues treated with MSCs^SC^-Exos or CAP-MSCs^SC^-Exos loading with FAM-miR-199a-3p. scale bar: 5 μm. **C** Schematic diagram of different MSCs^SC^-Exos delivery systems treatment strategy in DMM-induced OA mice. **D** The relative expression of miR-199a-3p in cartilage tissues treated with different MSCs^SC^-Exos delivery systems was detected by RT-PCR in DMM-induced OA mice. **E** Protein level of mTOR in cartilage tissues treated with different MSCs^SC^-Exos delivery systems was examined by western blotting in DMM-induced OA mice. **F** Safranin O/fast green staining of cartilage morphology in each group mice, scale bar: 500 μm (up) and 100 μm (down).**G** The OARSI scoring system quantify the severity of cartilage destruction. n = 6 for each group. **H** and **I** The immunohistochemistry analysis of COL2A1 and MMP13 in articular cartilage of OA mice model. n = 6 for each group. Scale bar: 50 μm. **J** Transmission electron microscopy (TEM) image of autophagic vesicles in cartilage of OA mice. The white arrow indicates the cell bilayer membrane structure of autophagic vesicles. Scale bar: 0.5 μm.* n* = 6. * p < 0.05, ** p < 0.01, *** p < 0.001, **** p < 0.001, ns not significant

Whether CAP-tagged exosomes can deliver miR-199a-3p within the target site in vivo were investigated. The DMM-induced mice model of OA were carried out. First, the MSCs^SC^-Exos/FAM-miR-199a-3p or CAP-MSCs^SC^-Exos/FAM-miR-199a-3p were injected into the cavity of surgical joint of the OA mice. The trial mouse were killed after 48 h, and the keen joints were fixed with 4% paraformaldehyde, decalcified, stained and investigated (Fig. 8A). The significantly higher FAM signal of cartilage was observed in the CAP-MSCs^SC^-Exos/FAM-miR-199a-3p treatment group than in control MSCs^SC^-Exos without the CAP peptide, and the FAM signal of CAP-MSCs^SC^-Exos/FAM-miR-199a-3p treatment group enriched in the deeper layer of articular cartilage (Fig. 8B). Meanwhile, QRT-PCR analysis of miR-199a-3p expression in the knee joint also indicated that the CAP-MSCs^SC^-Exos/miR-199a-3p delivery system significantly increased miR-199a-3p levers in cartilage as compared with the other group, including the group injected of MSCs^SC^-Exos without the CAP peptide (Fig. 8D). These results showed that the CAP sequence facilitates the penetration of MSCs^SC^-Exos to the cartilage, and effectively increased the levels of miR-199a-3p in cartilage.

Second, the DMM-induced OA mice were treated with CAP-MSCs^SC^-Exos/miR-199a-3p and MSCs^SC^-Exos/miR-199a-3p that diluted in PBS according to the strategy showed in (Fig. 8C). Briefly, MSCs^SC^-Exos preparations (1 × 10^10^particles/ml) were administrated to OA mice via intra-articular injections once every week for consecutive four weeks, and afterward the mice were sacrificed, and cartilage tissue was dissected for histological assessment. The results of Safranin O/Fast Green staining showed that, articular cartilage was obviously damaged after OA surgery; while the MSCs^SC^-Exos-treated groups exhibited improvement of cartilage impair (Fig. 8F). The both groups injected of CAP-MSCs^SC^-Exos/miR-199a-3p and MSCs^SC^-Exos/miR-199a-3p showed higer significantly improvement compared to the only MSCs^SC^-Exos-injected group in OA rats, especially CAP-MSCs^SC^-Exos/miR-199a-3p showed the most complete integration of cartilage with a smooth surface. The OARSI scoring including maximal and summed scores were taken to quantify the severity of tibial plateau lesion, and the results revealed that all the MSCs^SC^-Exos -treated groups impeded the cartilage lesion; especially the group injected of CAP-MSCs^SC^-Exos/miR-199a-3p showed the most significant effects after DMM surgery (Fig. 8G). Moreover, the change of chondrogenic proteins (COL2A1) and MMP13 expression of articular cartilage were assessed in OA rats, using IHC. The expression levels of proteins COL2A1 were increased in all the MSCs^SC^-Exos-treated groups comparing to the PBS group; especially the group injected of CAP-MSCs^SC^-Exos/miR-199a-3p showed the most significant effects after DMM surgery (Fig. 8H). The expression levels of MMP13 exhibited the opposite effects with the MSCs^SC^-Exos treatment (Fig. 8I).

Furthermore, we clarify the mechanism of the engineered MSCs^SC^-Exos on OA therapy focused on the autophagy of damaged cartilages, as the above results showed that MSCs^SC^-Exos promoted the repair of injured cartilage via increasing the levels of autophagy in OA model. The autophagosomes and autolysosomes of articular cartilage were determined by TEM assay, the gold standard to evaluate the autophagy. The representative photographs showed that the autophagic vesicles in cartilage of OA rats were increased in all the MSCs^SC^-Exos-treated groups comparing to the PBS group; especially the group injected of CAP-MSCs^SC^-Exos/miR-199a-3p showed the most significant effects after DMM surgery (Fig. 8J), indicating the most enhanced autophagy levels on the OA group injected of CAP-MSCs^SC^-Exos/miR-199a-3p. Then, the protein expression mTOR in OA cartilage were detected, as we revealed the MSCs^SC^-Exos enhanced the levels of autophagy in OA cartilage through downregulating the mTOR signal pathway. The WB results showed that the protein expression of mTOR in OA cartilage were remarkably decreased in all the MSCs^SC^-Exos-treated groups comparing to the PBS group; especially the group injected of CAP-MSCs^SC^-Exos/miR-199a-3p showed the most significant effects after DMM surgery (Fig. 8E). The protein expression of mTOR in OA cartilage are consistent to the former results of miR-199a-3p levels (Fig. 8D) and autophagy levels in OA cartilage of different groups (Fig. 8J). These above results presented that the excellent effect of engineering MSCs^SC^-Exos on the injured cartilage are related to the decreased protein expression of mTOR and enhanced levels of autophagy in OA cartilage, and the CAP-MSCs^SC^-Exos/miR-199a-3p showed the most significant effect.

Collectively, the above results revealed that MSCs^SC^-derived exosomes could promote chondrocyte autophagy to increase its catabolism and inhibit its anabolism, especially CAP-MSCs^SC^-Exos, which could be effectively delivered into deep articular tissues, then exert the protective effect on cartilage of OA animal model. To a deeper lever, these data also provided evidence that elevated miR-199a-3p induced by MSCs^SC^-Exos was of vital importance to cartilage destruction, and through the mTOR signal pathway in OA model. The intra-articular injection of engineered exosome derived from MSCs^SC^ with a target delivery of miR-199a-3p to chondrocytes holds promise as a safe and viable treatment for cartilage damage.

## Discussion

OA is recognized as a mild inflammatory disease, which affects the entire joint, especially progressive degradation of articular cartilage [1, 55–57]. Cartilage degradation is a crucial fingerprint in OA initiation, along with damaged cartilage homeostasis owing to excessive catabolism [58]. On a molecular level, increased levels of the matrix metalloproteinases (MMPs) along with decreased levels of chondrogenic proteins Collagen type II (COL2A1) are primarily accountable for the earliest osteoarthritic alterations [59]. MSCs-Exos therapy has been proven effective for cartilage repair in animal and clinical studies [10]. The previous study about the infrapatellar fat pad-derived MSCs, the other source of ADSCs, has reported the protective effect on damaged cartilage in an exosome-dependent manner [18]. Compared with the wide application of other types MSCs-Exos on OA disease, there are few studies on ADSC-Exos, especially subcutaneous fat (SC)-derived MSCs. In this research, we identified that human subcutaneous fat-derived MSCs, and isolated MSCs^SC^-Exos. The DMM and ACLT surgical models may result in gradual, progressive and site-specific articular cartilage deterioration, osteophytes, and subchondral bone abnormalities, were adopted in this study [32, 33]. Similar to the previous researches, our study showed that administration with MSCs^SC^-Exos could prevent cartilage damage and ECM degeneration, accompanied by the increased levels of COL2A1 and decreased levels of MMP-13 in OA cartilages.

Autophagy is an essential cellular homeostatic system that removes defective cellular organelles and macromolecules [60]. The cartilage is an aneural, avascular, alymphatic and viscoelastic connective tissue, and its nutrition and oxygen was supplied by the synovial fluid and subchondral bone [61]. The metabolism of chondrocytes and ECM is crucial to the overall cartilage integrity [62], as the cartilage tissues have low ability to self-repair and remodeling; autophagy serves a vital role in maintain cartilage homeostasis [63, 64]. Chondrocyte autophagy is particularly important in the initial and developmental phases of articular cartilage breakdown, and it decreases with OA progression [65, 66]. Previous studies showed that exosomes derived from infrapatellar fat pad MSCs (MSC^IPFP^-Exos) protect articular cartilage through modulating autophagy [18]; exosomes derived human umbilical cord mesenchymal stem cells (hUC-MSCs) inhibit microglia pyroptosis through promoting autophagy [67]. Taken together, these findings revealed that MSCs-Exos could exert a protective effect on the damaged cartilage through up-regulated autophagy, and targeting the autophagy of chondrocytes could be a promising strategy for OA therapy. Similar to the previous researches, our study showed that MSCs^SC^-Exos ameliorated the cartilage injury and ECM degeneration through enhancing the autophagy level of chondrocytes, demonstrated by the analysis the gene expression of cartilage samples in OA control group and MSCs^SC^-Exos treated group through the KEGG and GSEA enrichment. Additionally, the significantly increased expression levels of LC3B and the amount of autophagosome in OA cartilage and chondrocytes also presented the enhanced autophagy level in this study.

To explain the mechanism by which MSCs^SC^-Exos mediated the autophagy, we used RNA-seq to analyze the gene expression of cartilage samples in DMM + ACLT-induced OA control group and MSCs^SC^-Exos treated OA group, and the alteration of mTOR was the most noticeable among these differentially expressed genes. This study showed that MSCs^SC^-Exos treatment reduced the amout of mTOR protein and the related downstream signal pathway in the OA model. Notably, mTOR, a crucial negative regulator in autophagy process due to nutrient deprivation and stress, plays an important role in chondrocyte metabolism and OA pathogenesis [68]. Moreover, the mTOR pathway was active in OA development, along with the inhibited autophagy process [68]. Earlier research shown that MSC^IPFP^-Exos may greatly increase autophagy level in chondrocytes by inhibiting mTOR [18]; exosomes derived from bone mesenchymal stem cells (BMSC-Exos) relieve renal interstitial fibrosis via regulation of the mTOR signaling pathway and downstream autophagy [69]; Extracellular vesicles derived from bone mesenchymal stem cells (BMSC-EVs) can protect BMECs from damage by enhancing the autophagy levels through the mTOR pathway [70]. Taken together, these results suggested that MSCs-Exos can exert a protective effect on damaged cartilage through regulation of the mTOR signaling pathway thus enhance the autophagy process of chondrocyte, especially MSCs^SC^-Exos, which could directly inhibit mTOR expression, thus the increased autophagy to promote repairmen of destructed cartilage.

Exosomes are spherical membrane vesicles with lipid bilayer structures and embedded cellular elements, for instance, cytosolic proteins, lipids and nucleic acids (predominantly microRNAs) [13]. Exosomes function as a unique intercellular communication mechanism by transporting biological components between cells [13]. The miRNAs, enriched in exosomes, are noncoding RNAs that disrupt gene expression by binding to corresponding nucleotide of the target mRNA, usually in the 3ʹ untranslated region (UTR) [71]. Previous studies demonstrated that exosomes produced by miR-92a-3p-overexpressing hBMSCs could repair degraded cartilage via directly targeting Wnt5A on an OA mice model [72]; MSC^IPFP^-Exos protected articular cartilage and improved gait disorders through suppression of mTOR-autophagy pathway via mir-100-5p [18]; MSCs^SC^-Exos mitigated cartilage degradation by transferring miRNA-376c-3p and targeting the Wnt-bet-catenin signaling axis in MIA-induced OA model [20]. Taken together, it is necessary that miRNA produced from MSCs^SC^-Exos could take part in improving cartilage injury. Subsequently, we revealed that MSCs^SC^-Exos facilitated the exosomal mir-199a-3p deliver to OA cartilage through the enhanced mTOR-mediated autophagy in this study. MSCs^SC^-Exos treatment significantly upregulated the level of miR-199a-3p in vitro and in vivo, meanwhile, the expression of mir-199a-3p was highly decreased in OA progression as previously reported [48, 50]. Several miRNAs have been proposed as biomarkers for OA disease in previous research. It remains to be further confirmed whether mir-199a-3p in cartilage may serve as an indicator for OA disease. Our findings also revealed that mir-199a-3p enhanced the autophagy level of chondrocytes and ECM synthesis in vitro and in vivo. Previous researches showed that miR-199a-3p could facilitate chondrocyte proliferation, suppress chondrocyte apoptosis and play an anti-catabolic role in OA model [48, 49]. Therefore, mir-199a-3p may potentially be a target for OA, which need in-depth investigation. Our results also presented that miR-199a-3p was transferred from MSCs^SC^-Exos, and directly regulated the expression of mTOR in the recipient cells. Previous reports have shown the therapeutic effect of exosomal miR-199a-3p from MSCs on different diseases. BMSCs-Exos increased epithelial sodium channel (ENaC) expression through mir-199a-3p in acute lung injury [73]. BMSCs-Exos protect against renal ischemia/reperfusion injury via transferring mir-199a-3p in acute kidney injury [74]. These reports suggested that miR-199a-3p derived from MSCs-Exos was significant for cell-to-cell communication and regulating recipient cells function. Furthermore, we are the first to report that miR-199a-3p is implicated in subcutaneous fat derived MSCs-Exos in cartilage repair in OA progression.

To exert the superior effect of MSCs^SC^-Exos on the injured cartilage, we designed the targeted drug delivery systems (DDSs) in OA therapy based on the following features: (1) the excellent tissue penetration capability of exosomes through the densely packed ECM of the cartilage; (2) the comprehensive benefits of exosomes derived from MSCs^SC^; (3) the enhanced cartilage homeostasis and ECM regeneration with miR-199a-3p; (4) specially delivering the therapeutic agents (miR-199a-3p) to the targeted sites (chondrocytes). Then the engineered MSCs^SC^-Exos drug delivery systems based on MSCs^SC^-Exos modified with a chondrocyte-targeting peptide (CAP) loading with miR-199a-3p, named CAP-Exos^SC^/miR-199a-3p were constructed for OA treatment. CAP-Exos^SC^/miR-199a-3p could specifically deliver miR-199a-3p to cartilage through fusing peptide CAP with the exosomal membrane protein Lamp 2b, according to the previous reports [31, 37, 38]. Most published studies [31, 37, 38] about exosomes for cartilage regeneration or OA treatment are based dendritic cells and only showed single function, while the CAP-Exos^SC^/miR-199a-3p showed comprehensive functions, combining the merits of exosomes derived from MSCs^SC^ and the functions of cartilage protection from loading miR-199a-3p in OA therapy. To the best of our knowledge, this is the first study of drug delivery systems using exosomes produced by MSCs^SC^-derivatives, which are easily collective by liposuction and belong to surgical waste then could be used for allogeneic treatment without ethical issues on one hand, and could be used for autologous treatment without immunological rejection on the other hand.

There is a limited finding to this study worth noting. On the part, this research presented that MSCs^SC^-Exos could work through the exosomal miR-199a-3p, and the therapeutic effects of MSCs^SC^-Exos on OA were partially abolished with antagomir-199a-3p injections, indicating other molecules in MSCs^SC^-Exos may participate this beneficial effect. A great quantity of molecules, for instance, lipids, proteins or nucleic acids (mostly miRNAs) are embedded in exosomes, which may contribute to the therapeutic effects of MSCs^SC^-Exos [13], are need to depth pursued. For example, a previous study on the infrapatellar fat pad (IPFP)-derived MSC showed that MSC^IPFP^-Exos protected cartilage and improved gait impairments via transferring miR-100-5p through inhibiting mTOR in OA [18]. The miRNAs including: miR-199a-3p, miR-100-5p, miR-199a-3p, miR-99a-5p; are predicted potential miRNAs that target specifically the 3’UTR of mTOR mRNA using the online bioinformatics methods, which are also existed in subcutaneous fat MSCs derived exosomes. In the miRNAs expression profiles of subcutaneous fat MSCs derived exosomes in this study and previous report [75], miR-199a-3p and miR-100-5p are all most highly enriched miRNAs. We conclude that the two miRNAs of miR-199a-3p and miR-100-5p may participate in the therapeutic effect of MSCs^SC^-Exos in OA through inhibiting the mTOR signal pathway. Consistently, it is well known that a complex network of Exos-embedded miRNAs will be accountable for the potential therapeutics of MSCs^SC^-Exos in OA. On the other part, the complexity and technical challenges might limit the clinical transformation of CAP-Exos^SC^/miR-199a-3p as drug delivery systems at present, and more studies should be in-depth performed.

## Conclusion

In summary, a chondrocytes-targeted engineering exosomes CAP-Exos^SC^/miR-199a-3p presented the superior effect on impaired cartilage in OA therapy. First, the mechanism of MSCs^SC^ derived exosomes was clarified. MSCs^SC^-Exos could enriched in keen joint cartilage and deliver miR-199a-3p into chondrocytes. The enhanced miR-199a-3p expression in chondrocytes specifically targeted the 3ʹ UTR region of mTOR mRNA, resulting in the decrease of mTOR protein level and the related downstream signal pathway. The repressive mTOR signal also increased chondrocyte autophagy, which increased anabolism and suppressed catabolism in OA cartilage. MSCs^SC^-Exos partially alleviated the pathological severity through the miR-199a-3p-mediated mTOR-autophagy pathway in OA animal model. Then, CAP-Exos^SC^/miR-199a-3p were constructed, which took advantage of the intrinsic comprehensive benefits of MSCs^SC^-Exos and collaborated with the loaded miR-199a-3p to enhance the therapeutic effects on OA diseases via the engineering approach. As adipose tissue could be obtained easily in clinic, our findings could present a novel technique based on MSCs^SC^-Exos for OA therapy, and may lead to a prospective cell-free therapy that relies on the application of MSCs^SC^-Exos for drug delivery in the future.

### Supplementary Information

**Additional file 1: Figure S1.** The treatment effect of MHY1485 on cell viability of rat chondrocytes. **A** CCK-8 assay. Dosage effect with the treatment of MHY1485 at 25, 50, 100, 200 and 500 μmol/L concentration on cell viability of rat chondrocytes with 12h. **B** CCK-8 assay. Time effect with the treatment of MHY1485 at 0, 6, 12, 18, 24, 30, 36 h on cell viability of rat chondrocytes with a concentration of 50 μmol/L. The data are shown as the mean ± standard error (SEM), N = 3. * p < 0.05, **p < 0.01, ***p < 0.001, ****p < 0.001, ns not significant. **Figure S2. **Quantification of the loading efficiency of different Exos^SC^ preparations using electroporation.

## Data Availability

The datasets used and/or analyzed during the current study are available from the corresponding author on reasonable request.

## Abbreviations

OA: Osteoarthritis
ADSCs: Adipose-derived stromal cells
ECM: Extracellular matrix
DMM: Destabilization of the medial meniscus
ACLT: Anterior cruciate ligament transection
qRT-PCR: Quantitative real time-polymerase chain reaction
IF: Immunofluorescence
MSCs: Mesenchymal stem cells
MicroRNAs: MiRNAs
SD rats: Sprague–Dawley rats
PBS: Phosphate-buffered saline
FBS: Fetal bovine serum
OARSI: Osteoarthritis Research Society International
DAPI: 4′, 6-diamidino-2-phenylindole
ORF: Complete open reading frame
*IL-1β*: Interleukin-1 beta
U6: U6 small nuclear RNA
SDS-PAGE: Sodium dodecyl sulfate–polyacrylamide gel electrophoresis
PVDF: Polyvinylidene fluoride
COL2A1: Chondrogenic proteins Collagen type II
MMPs: The matrix metalloproteinase
LC3: Microtubule associated protein 1 light chain 3
p-p70S6: Phosopho-70-kDa S6 ribosomal protein
ANOVA: One-way analysis of variance
