# Supplementary material for: Engineering exosomes derived from subcutaneous fat MSCs specially promote cartilage repair as miR-199a-3p delivery vehicles in Osteoarthritis
Source: J Nanobiotechnology. 2023 Sep 22;21:341. doi: 10.1186/s12951-023-02086-9 (PMC10515007; doi:10.1186/s12951-023-02086-9)

**Figure S1.** The treatment effect of MHY1485 on cell viability of rat chondrocytes.

**A.** CCK-8 assay. Dosage effect with the treatment of MHY1485 at 25, 50, 100, 200 and 500 μmol/L concentration on cell viability of rat chondrocytes with 12h.

**B.** CCK-8 assay. Time effect with the treatment of MHY1485 at 0, 6, 12, 18, 24, 30, 36h on cell viability of rat chondrocytes with a concentration of 50 μmol/L.

The data are shown as the mean ± standard error (SEM), N=3. * *p*<0.05, ** *p*<0.01, *** *p*<0.001, **** *p*<0.001, ns not significant.

**A. B.**


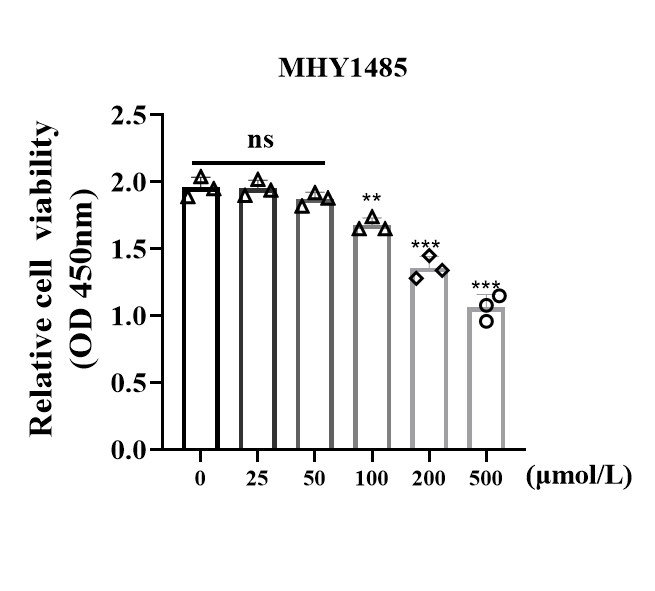

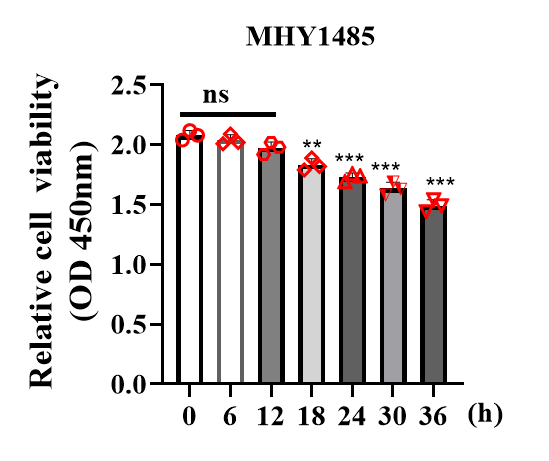


**Figure S2.** Quantification of the loading efficiency of different Exos^SC^ preparations using electroporation.


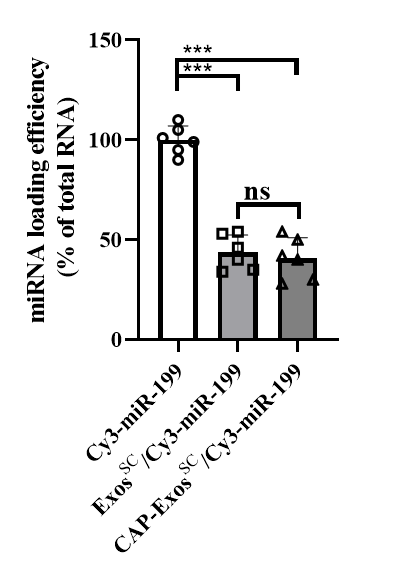

Supplement: Supplementary file 1 — Additional file 1: Figure S1. The treatment effect of MHY1485 on cell viability of rat chondrocytes. A CCK-8 assay. Dosage effect with the treatment of MHY1485 at 25, 50, 100, 200 and 500 μmol/L concentration on cell viability of rat chondrocytes with 12h. B CCK-8 assay. Time effect with the treatment of MHY1485 at 0, 6, 12, 18, 24, 30, 36 h on cell viability of rat chondrocytes with a concentration of 50 μmol/L. The data are shown as the mean ± standard error (SEM), N = 3. * p < 0.05, **p < 0.01, ***p < 0.001, ****p < 0.001, ns not significant. Figure S2. Quantification of the loading efficiency of different ExosSC preparations using electroporation. [file 12951_2023_2086_MOESM1_ESM.docx]
